# Estuarine mangrove niches select cultivable heterotrophic diazotrophs with diverse metabolic potentials—a prospective cross-dialog for functional diazotrophy

**DOI:** 10.3389/fmicb.2024.1324188

**Published:** 2024-05-24

**Authors:** Sumana Mondal, Biswajit Biswas, Rajojit Chowdhury, Rudranil Sengupta, Anup Mandal, Hemendra Nath Kotal, Chayan Kumar Giri, Anjali Ghosh, Subhajit Saha, Mst Momtaj Begam, Chandan Mukherjee, Ipsita Das, Sandip Kumar Basak, Mahashweta Mitra Ghosh, Krishna Ray

**Affiliations:** ^1^Environmental Biotechnology Group, Department of Botany, West Bengal State University, Kolkata, India; ^2^Department of Microbiology, St. Xavier’s College (Autonomous), Kolkata, India; ^3^Department of Botany, Sree Chaitanya College, Habra, India; ^4^Department of Botany, Kalimpong College, Darjeeling, India; ^5^School of Biological and Life Sciences, Galgotias University, Greater Noida, India; ^6^Department of Botany, Sarat Centenary College, Dhaniakhali, India

**Keywords:** biological nitrogen fixation, free-living heterotrophic diazotroph, estuarine mangrove ecosystem, Indian Sundarbans, stringent narrow niche, multidimensional specialization

## Abstract

**Introduction:**

Biological nitrogen fixation (BNF), an unparalleled metabolic novelty among living microorganisms on earth, globally contributes ~88-101 Tg N year^−1^ to natural ecosystems, ~56% sourced from symbiotic BNF while ~22-45% derived from free-living nitrogen fixers (FLNF). The success of symbiotic BNF is largely dependent on its interaction with host-plant, however ubiquitous environmental heterotrophic FLNFs face many limitations in their immediate ecological niches to sustain unhindered BNF. The autotrophic FLNFs like cyanobacteria and oceanic heterotrophic diazotrophs have been well studied about their contrivances acclimated/adapted by these organisms to outwit the environmental constraints for functional diazotrophy. However, FLNF heterotrophs face more adversity in executing BNF under stressful estuarine/marine/aquatic habitats.

**Methods:**

In this study a large-scale cultivation-dependent investigation was accomplished with 190 NCBI accessioned and 45 non-accessioned heterotrophic FLNF cultivable bacterial isolates (total 235) from halophilic estuarine intertidal mangrove niches of Indian Sundarbans, a Ramsar site and UNESCO proclaimed World Heritage Site. Assuming ~1% culturability of the microbial community, the respective niches were also studied for representing actual bacterial diversity via cultivation-independent next-generation sequencing of V3-V4 rRNA regions.

**Results:**

Both the studies revealed a higher abundance of culturable Gammaproteobacteria followed by Firmicutes, the majority of 235 FLNFs studied belonging to these two classes. The FLNFs displayed comparable selection potential in media for free nitrogen fixers and iron-oxidizing bacteria, linking diazotrophy with iron oxidation, siderophore production, phosphorus solubilization, phosphorus uptake and accumulation as well as denitrification.

**Discussion:**

This observation validated the hypothesis that under extreme estuarine mangrove niches, diazotrophs are naturally selected as a specialized multidimensional entity, to expedite BNF and survive. Earlier metagenome data from mangrove niches demonstrated a microbial metabolic coupling among C, N, P, S, and Fe cycling in mangrove sediments, as an adaptive trait, evident with the co-abundant respective functional genes, which corroborates our findings in cultivation mode for multiple interrelated metabolic potential facilitating BNF in a challenging intertidal mangrove environment.

## 1 Introduction

Biological N_2_ fixation (BNF; i.e., diazotrophy) is an important metabolic pathway that occurs in living organisms and contributes ~88–101 Tg N year^−1^ to global natural ecosystems (Davies-Barnard and Friedlingstein, 2020). Symbiotic diazotrophs contribute ~56% while free-living N_2_ fixers (FLNF) contribute ~22–45% of it (Davies-Barnard and Friedlingstein, 2020). Autotrophic cyanobacteria (blue-green algae) are well-known FLNF. However, non-cyanobacterial diazotrophic bacteria and archaea, which are eco-physiologically variable and ubiquitous in natural habitats, also have a high potential to contribute to global BNF. Being largely dependent on heterotrophic nutrition, these diazotrophs are categorized as heterotrophic diazotrophs (Bombar et al., 2016). Based on metagenome data on diazotrophic populations from open oceans and seas, heterotrophic diazotroph non-cyanobacteria are more widespread than diazotrophic cyanobacteria (Delmont et al., 2022), belonging to wide-ranging bacterial taxa (comprising Alphaproteobacteria, Betaproteobacteria, Deltaproteobacteria, Gammaproteobacteria, and Firmicutes) (Smercina et al., 2019).

In natural ecosystems, symbiotic and non-symbiotic diazotrophs face similar constraints that limit BNF efficiency as well as factors that positively regulate BNF activity (Smercina et al., 2019; Yang et al., 2022). Among the limitations, individual deficiencies or co-deficiencies of Mo, Fe, P, or V in microhabitats primarily decrease BNF, while increased P, Fe, Mo, and V availability increase BNF (Smercina et al., 2019). Mo supplementation in multiple locations (from tropical forests to boreal environments) increased FLNF, while N supplementation completely suppressed FLNF (Dynarski and Houlton, 2018). Additionally, during BNF, Fe/P use efficiencies under P limitation or Fe/P co-limitation were improved by environmental sensing mechanisms in the marine diazotrophic cyanobacterium *Crocosphaera watsonii* (Yang et al., 2022). On the other hand, bioavailable N (in the form of ammonium, glutamine, glutamate, or nitrate) decreased FLNF-mediated BNF by downregulating the *nifA* gene (which encodes a *nif*-specific regulatory protein) without affecting the activity of already-synthesized nitrogenase (the unique essential enzyme of BNF) in most diazotrophs (Smercina et al., 2019). N limitation was a pre-requisite for maintaining diazotrophy in surface waters, with physiological feedback inhibition of nitrogenase by dissolved inorganic N (such as ammonium or nitrate) causing non-diazotroph overpopulation (Mills et al., 2004). In addition, despite the dynamic status of rhizospheric O_2_ or dissolved O_2_ (DO) in waterbodies, nitrogenase activity in aerobic organisms is often suppressed at high O_2_, as O_2_ irreversibly inhibits nitrogenase (Smercina et al., 2019). For heterotrophic FLNF, available C sources are also crucial for BNF. BNF is an energetically expensive process, heavily dependent on available ATP and its subsequent expenditure. Ideally, 16 ATPs need to be invested per N_2_ and 2H^+^ reduced to 2NH_3_ by nitrogenase (Rascio and La Rocca, 2008). Thus, because of its high energy cost, BNF is only induced in the absence/depletion of all bioavailable N forms (Rascio and La Rocca, 2008). At the rhizospheric level, plant root exudates (including sugars, organic acids, and mucilage) or overall soil organic C content, can act as C sources for heterotrophic FLNF (Smercina et al., 2019). BNF by heterotrophic FLNF was positively associated with total organic C content (Smercina et al., 2019; Geisler et al., 2022), but non-diazotrophs may outcompete these heterotrophic FLNF in C- and N-replete niches (Sun et al., 2020). Also, the relative abundance of the *nifH* gene (which encodes the iron protein subunit of nitrogenase) in native niches was negatively associated with total N or total organic C content in the niche, indicating loss of the diazotrophic function in niches with high C and N availability (Sun et al., 2020).

FLNFs have evolved several biochemical/metabolic strategies to overcome adverse factors to continue cellular BNF uninterrupted. Under P limitation, diazotrophs upregulate *pstS* (which encodes a high-affinity phosphate transporter) and shift to using soluble P by upregulating *phoA/B* and *phoX* (to produce alkaline phosphatases) (Yang et al., 2022; Zhu et al., 2023). Fe limitation in diazotrophic photosynthetic cyanobacteria downregulates Fe-rich photosystem I protein complexes, upregulates the chlorophyll-binding Fe-free protein IsiB (which forms light-harvesting antennae), and replaces the Fe-containing ferredoxin with the Fe-free flavodoxin IsiB for photosynthetic electron transfer (Yang et al., 2022; Zhu et al., 2023). The diazotrophic cyanobacterium *Crocosphaera* sp. overcomes Fe limitation by ensuring diurnal photosynthesis and nocturnal BNF, judiciously shuttling cellular Fe for the synthesis of Fe proteins required for both these metabolic processes (Yang et al., 2022). In addition, it adopts a strategy of rapid growth, reduced cell size, and a resource-competent phenotype in response to Fe/P co-limitation (Yang et al., 2022). In filamentous free-living cyanobacteria, nitrogenase, which is sensitive to O_2_, is produced in O_2_-impermeable heterocysts, which thereby spatially separates nitrogenase from O_2_-evolving photosynthesis (Kumar et al., 2010). In unicellular *Crocosphaera watsonii*, *nifH* expression peaks at night, protecting nitrogenase from the O_2_ produced in daytime photosynthesis (Bombar et al., 2016). Marine diazotrophs may conduct BNF under high O_2_ by synthesizing extracellular organic polymer matrices to produce a pellicle-like structure or alginate capsule around their cells, as observed for non-marine *Pseudomonas* spp. and *Azotobacter vinlandii* (Bombar et al., 2016). Marine diazotrophs also form large aggregates of cells (>1 mm in diameter) as an innovative strategy to create low-O_2_ microhabitats near the center of the aggregates (Bombar et al., 2016; Geisler et al., 2022). These aggregates are typified by high labile C (originating from the hydrolysis of the aggregates’ polysaccharide matrix) and N limitation (Geisler et al., 2022). Rhizospheric soil texture also influences BNF. BNF increases in rhizospheric soils with greater clay content, and >70% of the FLNF populations are located in micro-aggregates of soil particles (>50 mm in diameter). Clay soils (compared to sandy soils) supported nitrogenase activity to a greater degree by shielding FLNF microhabitats from external O_2_ (Gupta and Roper, 2010; Smercina et al., 2019). In addition, regarding other relevant metabolic adaptations, several heterotrophic FLNF have evolved the following metabolically diverse pathways (besides aerobic respiration) to gain ATP for energy-demanding BNF: anoxygenic photosystem II, thiosulfate oxidation, dissimilatory nitrate reduction to ammonia, and dissimilatory sulfate reduction (Delmont et al., 2022). Even acidophilic lithoheterotrophic diazotrophic iron-oxidizing bacteria (IOB; such as *Acidithiobacillus ferrooxidans*, *Thiobacillus ferrooxidans*, and *Leptospirillum ferrooxidans*) have been reported (Norris et al., 1995; Parro and Moreno-Paz, 2004). Nitrate-reducing ferrous iron oxidation under both aerobic and anaerobic conditions indicates that denitrification is coupled with ferrous iron oxidation in these lithoheterotrophic IOB (which comprise up to 0.8% of all nitrate-reducing microbes) (Straub et al., 2001). Intriguingly, denitrification using NO_3_, NO_2_, or N_2_O as the terminal electron acceptor has been observed among heterotrophic diazotrophs (including *Pseudomonas* spp., *Azospirillum* spp., *Bradyrhizobium* spp., *Rhizobium* spp., and *Rhodopseudomonas* spp.) (Chan, 1985). This enigmatic co-occurrence of BNF and denitrification is advantageous for diverse heterotrophic diazotrophs in high-nitrate, low-O_2_ oceanic niches (Reeder, 2021; Reeder et al., 2022).

The intertidal mangrove ecosystem is a complex dynamic nutrient-limited ecosystem, largely influenced by the tidal flooding frequency, temperature, solar radiation, salinity, sediment texture, soil chemistry, and above all, fluctuating compositions of native nutrient-cycler microbial communities. Intertidal non-rhizospheric and rhizospheric niches in mangroves are often N-, P-, and Fe-deficient anoxic niches rich in sulfides (Alongi et al., 1992, 2002; Alongi, 2010; Reef et al., 2010; Almahasheer et al., 2016). In the presence of O_2_, reactive iron binds to inorganic P, which is adsorbed to the sediment, limiting P availability and decelerating P release from sublittoral sediments (Alongi, 2010; Reef et al., 2010). Iron is also precipitated (in forms such as pyrite) with free sulfides, creating an iron-limited environment (Alongi, 2010). The high denitrification rates remove the nitrate and nitrite pools from the intertidal niches, while concurrent high ammonification and BNF rates lead to simultaneous ammonia-N enrichment (Alongi et al., 2002; Reef et al., 2010). NH_4_^+^ is the dominant N-form in mangrove soils because of higher rates of ammonification to other N transformation processes existing in mangrove sediments (Reef et al., 2010; Alongi, 2013, 2020). In addition, ammonium adsorption to the sediments decreases due to the higher affinity of other seawater cations to be adsorbed, resulting in the free ammonium-N being the principal available form of N in mangrove shoreline environments (Reef et al., 2010). In mangrove niches, a more or less balanced N-budget (Alongi et al., 1992; Alongi, 2020) was demonstrated where net immobilization of NH_4_^+^ in organic-N was found to be the largest, estimated as the difference between gross and net ammonification and nitrification and the maximum of N being stored in sediments. Denitrification occurs mostly from surface soils (5–20 cm) and is the largest loss of N, equating to 10–35% of total N input (Alongi, 2013, 2020) while BNF is found to be ≤5% of total N input (Alongi, 2013). In deep intertidal sediments (~1 m deep), sulfate-reducing bacteria reduce Fe to forms that are unsuitable for binding to P, leaving some soluble P available. Nevertheless, in deep anoxic rhizospheric sediments (~1 m deep), sulfate reduction coincides with active BNF, in addition to the BNF observed in shallow rhizospheric sediments (~5–20 cm deep) (Alongi et al., 2002; Reef et al., 2010). In addition, a high BNF rate via diazotrophs was also observed from the surface of logs, barks, tree stems, cyanobacterial mats, aboveground roots, fresh and senescent leaves, and litter (Alongi, 2013, 2020).

In such a complex niche of high stringency and dynamicity, the following three variable N transformation pathways may operate: net denitrification (depending on nitrate and nitrite availability) including anaerobic ammonium oxidation, net ammonification (by microbial decomposers acting on organic N of the detritus/litter pool or dissimilatory reduction of nitrates), and net active BNF (at both near-surface and deeper regions of rhizospheric and non-rhizospheric niches in intertidal mangrove habitats and other associated micro-niches referred to earlier) (Reef et al., 2010; Alongi, 2013, 2020).

This present study is centered on estuarine intertidal mangrove niches of Indian Sundarbans, a Ramsar site^1^ and UNESCO-proclaimed World Heritage Site.^2^ Indian Sundarbans mangrove niche exhibited a pattern of N-cycling very similar to mangrove sediments reported worldwide (Ray et al., 2014). The surface sediment (up to 60 cm depth) exhibited the highest availability of ammonium-N, followed by nitrates and nitrite-N, ammonium being 12–18 times more abundant. Sediment pore water also possessed ammonium-N as the most abundant N form. BNF in sediments was found to be heavily dependent on diazotrophic bacteria, which showed higher BNF activity in October and April. Indian Sundarbans sediments are reported to be similarly N-deficient as observed across the study sites in Prentice, Lothian, Eco Camp, Bonnie camp, and Halliday island, having stored N-content even lower than those of mean marine sediments and Australian mangrove sediments (Ray et al., 2014). The characteristic traits of N-budget in Sundarban mangrove estuarine ecosystem can be stated as follows: (1) it acts as a sink for atmospheric N indicating net N biosphere-atmosphere exchange of different N forms; (2) it acts as a potential store for N-forms in sediments via absorption from tidal fluxes, retaining only 0.2% of the annual riverine transport; (3) major available N-sources are recycled within the biomass, sediments, litters, and atmosphere and the loss of N was found to be 22–23% of the inputs from the external sources; (4) BNF by diazotrophic bacteria (autotroph or heterotroph) had been an integral part of this N-cycling process in sediments (Ray et al., 2014).

The eastern part of Indian Sundarbans is protected under the Sundarbans Tiger Reserve^3^ and except for the campsites, other core areas are not accessible to researchers. Therefore, this study was restricted to the intertidal study sites of only the western part of the Indian Sundarbans for sediment core collections and evaluation of associated sedimentary geochemistry and prevailing hydrology along with both cultivation-dependent and cultivation-independent bacterial profiles in the sediments. In a cultivation-dependent analysis, we isolated 299 bacterial isolates [all accessioned in the National Center for Biotechnology Information (NCBI) database] and 45 non-accessioned isolates from below-ground estuarine mangrove niches from 2015 to 2020. The 299 accessioned bacterial isolates comprised 78 endophytes from mangrove root/pneumatophore endospheres (MRP_E_), 90 isolates from halophytic native grass rhizospheres in mangrove habitats (HNG_R_), 84 isolates from mangrove species rhizospheres (M_R_), and 47 isolates from cultivated rice rhizospheres (CR_R_) near Indian Sundarbans shoreline mangrove fringes (Supplementary Data). In settlement villages in the Indian Sundarbans, nearby lowland cultivated rice fields are juxtaposed with the shoreline mangrove fringes; they are almost confluent with the mangroves and are transitional regarding many parameters. CR_R_s have a high probability of having an estuarine mangrove niche edge effect. MRP_E_ are hubs of endophytes that migrate from mangrove intertidal rhizospheric niches, epitomizing the root–rhizosphere interaction continuum. Hence, in addition to M_R_ and HNG_R_, which are integral intertidal niches of estuarine mangrove habitats, we considered MRP_E_ and CR_R_ as relevant sources of culturable heterotrophic FLNF.

However, this cultivation-dependent study represented only 1% of the actual bacterial community composition for a particular niche (Bakken, 1997; Vieira and Nahas, 2005; Martiny, 2019). Hence, simultaneous cultivation-independent analyses of 16S rRNA gene abundances for the sediment cores were also undertaken from the HNG_R_, M_R_, and CR_R_ study sites to demonstrate existing actual bacterial diversity and abundances (within 1–37%). This Next Generation Sequencing (NGS) of 16S rRNA profiling was based on using the primer pair 341F-785R (V3-V4) that was reported to work more efficiently over other short amplicons targeting different variable regions (V-regions) of 16S rRNA such as V1–V2, V1–V3, V4, V4–V5, V6–V8, and V7–V9 irrespective of the reference databases and bioinformatic settings on taxonomic assignment used (Klindworth et al., 2013; Thijs et al., 2017; Rausch et al., 2019; Abellan-Schneyder et al., 2021; Katiraei et al., 2022). The primer pair 341F-785R from the V3-V4 region was found to represent an unbiased concurrent highest coverage of all operational taxonomic units (OTUs) under the domain Bacteria (96.1%), across several microbiomes like human gut microbiome (Abellan-Schneyder et al., 2021), for soil and plant-associated bacterial microbiomes (Thijs et al., 2017) and 10 different holobiont microbiomes ranging from basal aquatic metazoans to marine and limnic cnidarians, standard vertebrate and invertebrate model organisms to *Homo sapiens* in addition to the representative plant model species wheat (Rausch et al., 2019). These findings formed the basis of available commercial kits for 16S rRNA profiling analysis using the V3-V4 primers for PCR amplicon generation (López-Aladid et al., 2023).

We hypothesize that the stringent narrow parameters of intertidal mangrove niches of Indian Sundarbans may select for native heterotrophic FLNFs with co-occurring diverse metabolic functions that could aid the BNF process, and may represent a strategy to overcome adversities and thereby maintain BNF uninterrupted. Our objective was to analyze all the developed cultivable bacterial isolates (299 accessioned and 45 non-accessioned isolates as mentioned earlier), selected in abiotically stressed/nutrient-constrained estuarine mangrove niches, and screen them initially for their BNF attributes and subsequently evaluate them for their multiple interrelated primary BNF-facilitating metabolic functions like P solubilization, siderophore production, iron oxidation, denitrification, soluble P uptake, and poly-P accumulation under optimal laboratory conditions. The co-occurrence of all these functions if observed in many isolates in this study, may not indicate a chance phenomenon, rather it could validate our above-stated hypothesis.

## 2 Materials and methods

### 2.1 Sampling of estuarine mangrove sediments and mangrove species roots/pneumatophores from different study sites

Sediment cores (30 cm long and 4 cm wide) from depths of 0–15 cm and 15–30 cm were collected from 13 sites (5–25 per site) of the western part of the Indian Sundarbans. There were four sediment types: mangrove species rhizospheres (M_R_), halophytic native grass (such as *Porteresia coarctata*, *Myrostachya wightiana*, *Sporobolus virginicus*, and *Paspalum vaginatum*) rhizospheres in mangrove habitats (HNG_R_), cultivated rice rhizospheres near mangrove habitats (CR_R_), and mangrove non-rhizosphere sediments (M_NR_) (Supplementary Data). The name of the sites and their location co-ordinates are detailed (Supplementary Data) as well as a map of the sites/points sampled has been included in the Supplementary Figure S1. However, analyses described in this study were mainly limited to the surface sediments (0–15 cm). Segments of roots and pneumatophores of several mangrove and mangrove-associate species were also collected from the referred study sites (Supplementary Data; Supplementary Figure S1) The samples were brought to the laboratory in sterile bags maintained at 4°C for analysis. Composite soil samples were obtained from depths of 0–30 cm for each of the four sediment types, and the samples were then subjected to next-generation sequencing (NGS) of the V3–V4 region of the 16S rRNA.

### 2.2 Physical and nutrient parameters of sediments

Electrical conductivity (Chemiline CL250, Labline Technology Pvt. Ltd., Ahmedabad, India) (Janzen, 1993; Rhoades, 1996), pH (HANNA HI98319), temperature (HANNA Soil Test HI98331), and soil texture estimations (sand%, silt%, and clay%) (Kettler et al., 2001) were majorly assessed from 0–15 cm sediment cores. Next, exchangeable Na^+^ and K^+^ contents were measured by subjecting air-dried soil to extraction with 1 N ammonium acetate solution (pH 7.0) followed by incubation for 24 h for Na^+^ and K^+^ assessment using a flame photometer (Frontline, India) calibrated with 0–100 mg L^−1^ standard NaCl and KCl solutions (Toth and Prince, 1949). Organic C was measured using the acidified dichromate digestion method (Datta et al., 1962). Ammonia-N was extracted from the soil using 2 M KCl (Dorich and Nelson, 1983) and assessed using the phenate method (Park et al., 2009). Soluble P was extracted from the soil using a modified Morgan solution (McIntosh, 1969) and quantified using the molybdenum blue method (Krishnaswamy et al., 2009). Nitrate-N was extracted from the soil (Dorich and Nelson, 1983) and quantified using ultraviolet spectroscopy (Edwards et al., 2001). All these methods were based on spectrophotometry using a SmartSpec Plus spectrophotometer (Bio-Rad, CA, United States).

### 2.3 On-site hydrology assessment

The sites of on-site hydrology assessment are mentioned in Supplementary Data and Supplementary Figure S1. The salinity and pH of riverine water samples were tested using HANNA HI98319 and HI98107 portable meters, respectively. The salinity tester was calibrated using a 35.00 ppt (parts per thousand) calibration solution, while the pH meter was calibrated using pH 4.01 and pH 7.01 buffer solutions as per the manufacturer’s instruction before taking sample readings. Water samples’ turbidity was measured using the HANNA HI 98703 portable Turbidimeter. This instrument measures turbidity in the Nephelometric Turbidity Unit (NTU). This meter was calibrated as per the manufacturer’s manual using a four-step method with provided calibration solutions of 0.10 NTU, 15 NTU, 100 NTU, and 750 NTU. Instruments were calibrated every time before taking any measurements to ensure accurate results. The dissolved oxygen levels of water samples were measured using the HANNA HI914604 portable dissolved oxygen meter. The two-step calibration method (zero calibration, 0%, and slope calibration, 100%) was performed to calibrate the DO meter as per the manufacturer’s protocols.

### 2.4 Isolation via differential selection, identification, and colony-forming unit estimation of culturable native nutrient cycler bacteria

To isolate mangrove root endophytic bacteria from mangrove root/pneumatophore endospheres (MRP_E_), roots and pneumatophores were washed with tap water, cut into small pieces (2–5 mm) with a sterile blade, surface sterilized with 0.1% HgCl_2_, and inoculated in lysogeny broth overnight at 37°C (Boruah, 2020). The enriched culture was spread on YMI stringent minimal medium, i.e., iron-oxidizing bacteria (IOB) selection medium (IOM), to select IOB (Ghosh et al., 2014). The enriched culture was also spread on FLNF selection medium (FLNFM) (Thatoi et al., 2012) simultaneously to select for FLNF. The selected single colonies were tested for purity by Gram staining (Bartholomew and Mittwer, 1952; Fall, 2000).

Similarly, to isolate bacteria from the four sediment types, serially diluted soil samples from 0 to 15 cm sediment cores were plated on IOM or FLNFM to establish pure cultures, which were repeatedly streaked onto the same media to confirm the metabolic identity as IOB or FLNF.

Genomic DNA was isolated (Moore et al., 1999, 2004; Mahansaria et al., 2015) from selected pure cultures from the CR_R_, M_R_, HNG_R_, M_NR_, and MRP_E_ groups. Next, PCR amplification of partial 16S rRNA (1.5 kb amplicon size) was carried out using a standard procedure involving universal primers 27F and 1492R (Frank et al., 2008; Galkiewicz and Kellogg, 2008). The amplicons were partially sequenced. The sequences were then subjected to BLAST search (using blastn suite of NCBI) for identification. They were then submitted to the NCBI database and granted accession numbers by NCBI (Supplementary Data).

For CFU estimation of various nutrient cyclers in the sediments, 1 g soil was suspended in 10 mL sterile physiological saline (0.85% NaCl), serially diluted up to 10^5^ times, and plated onto the following differential growth selection media for incubation for 8–10 days at 30–32°C: media for ammonifying bacteria (AB) (Rakshit et al., 2017), nitrifying bacteria (NB) (Elbanna et al., 2012), and denitrifying bacteria (DB) (Wu et al., 2013), NBRIP medium for phosphate-solubilizing bacteria (PSB) (Nautiyal, 1999), and Jensen’s medium for FLNF (Sahadevan et al., 2016).

### 2.5 Iron oxidation assay

Both IOM and FLFNM selected isolates (IOBs already repeatedly selected on IOB stringent minimal medium of Ghosh et al., 2014) were inoculated in the same medium at a pH of 6.0 (maintained throughout by 10 mM 2-(N-morpholino)ethanesulfonic acid (MES) buffer) in YMI broth with added 100 mg L^−1^ NO_3_^−^-N. The isolates were left to grow anoxically (using liquid paraffin oil on top of the broth) at an initial DO of 0.05 mg L^−1^ or aerobically at an initial DO of 6.85 mg L^−1^, undisturbed at 37°C. The IOB isolates were evaluated for iron oxide formation under both aerobic and anoxic conditions with 100 mg L^−1^ NO_3_^−^-N being added to the stringent minimal IOM at pH 6.0 to observe nitrate-dependent oxidation of ferrous iron under both aerobic and anaerobic conditions, which has been reported for DB (Straub et al., 2001). Fe^3+^ produced by the isolates in the medium was confirmed by adding 1 M ammonium thiocyanate solution in a 3:1 (v/v) ratio, which led to a brick-red precipitate (often within 15 min). The number of days required to detect Fe^3+^ (via oxidation of Fe^2+^) was recorded. DO levels were measured using a HANNA DO meter (model HI9146-04). The DO meter calibration was performed as per the manufacturer’s protocols and also detailed for this assay in the Supplementary material.

### 2.6 Acetylene reduction assays for the function of nitrogenase

Bacterial isolates repeatedly selected on FLNFM were inoculated in another nitrogen-free FLNFM (Watanabe et al., 1979; Baldani et al., 2014) at pH 7.0 and incubated for 72 h at 30°C. Next, 100 μL of the bacterial isolates were added to gas chromatography vials (with cotton plugs) containing N-free semi-solid medium (0.385% agar) and incubated for 72 h at 30°C. For the acetylene reduction assays, 1 mL acetylene gas (1,000–2,000 mg L^−1^) was injected into each vial, which was sealed with a rubber septum in place of the cotton plug and incubated at 29°C for 24 h. Reduction of acetylene (C_2_H_2_) to ethylene (C_2_H_4_), indicative of a functional nitrogenase complex, was measured based on nmol of ethylene generated in 24 h; an ethylene standard was also used (Kifle and Laing, 2016). The assay involved using a gas chromatograph (Agilent 6000 series) fitted with an HP-5 ms Capillary Column (50 m × 0.53 mm × 10 μm) at run time of 10 min and with an oven temperature of 150°C. Additionally, 100 μL from the same culture tested in the acetylene reduction assays was spread onto FLNFM agar (Watanabe et al., 1979; Baldani et al., 2014) and CFUs were recorded.

### 2.7 Phosphate solubilization assay

FLNFM- or IOM-selected isolates were repeatedly selected in phosphate-solubilizing NBRIP broth (Nautiyal, 1999), involving 5-day incubation at 30°C. The supernatant was collected and soluble P was quantified using the spectrophotometric molybdenum blue method, measuring the phosphomolybdate complex at 660 nm (Krishnaswamy et al., 2009) using a SmartSpec Plus spectrophotometer (Bio-Rad, CA, United States).

### 2.8 Siderophore production assay

FLNFM- or IOM-selected isolates were grown in lysogeny broth and then inoculated in standard iron-free succinate medium for 24–30 h at 28°C. Next, 100 μL supernatant was mixed with 900 μL chrome azurol S (CAS) assay solution (Fe–CAS–surfactant ternary complex) (Andrews and Duckworth, 2016) and incubated for 1 h. The color changed from blue to yellow, and the absorbance was measured at 630 nm (model UV-1800, SHIMADZU EUROPA GmbH). Siderophore production% was estimated based on the absorption maxima shift, as follows: ((Ar - As)/Ar) × 100, where Ar is the absorbance of the reference (CAS assay solution + uninoculated medium) and As is the absorbance of the sample (CAS assay solution + cell-free supernatant) (Jenifer et al., 2015).

### 2.9 Denitrification assay

Among the screened 235 culturable heterotrophic FLNF isolates (190 accessioned and 45 non-accessioned), we analyzed a representative subset of 24 accessioned isolates (belonging to a range of bacterial taxa) that were selected on both IOM and FLNFM. These 24 bacterial isolates and control bacterial strains (*E. coli* K12 ER2925 and *E. coli* K12 PR1031; New England Biolab) were inoculated in nitrate broth with NaNO_3_ equivalent to 100 mg L^−1^ NO_3_^−^-N and an initial pH of 7.0. They were incubated at 37°C for 24 h, anoxically (using liquid paraffin oil on top of broth) at an initial DO of 0.07 mg L^−1^ or aerobically at an initial DO of 6.78 mg L^−1^. The supernatant was spectrophotometrically (model UV-1800, SHIMADZU EUROPA GmbH) assessed for NO_3_^−^-N at 420 nm (absorption peak of nitrosalicylic acid) (Cataldo et al., 1975). The DO levels were regularly measured using a HANNA DO meter (model HI9146-04).

### 2.10 P uptake, poly-P accumulation, and visualization of poly-P granules

To assess P uptake, the abovementioned 24 bacterial isolates and control bacterial strains (*E. coli* K12 ER2925 and *E. coli* K12 PR1031; New England Biolab) were inoculated in a medium containing 500 mg L^−1^ PO_4_^3−^-P in the form of di-potassium hydrogen phosphate (K_2_HPO_4_) with an initial pH of 7.0. They were left at 37°C for 24 h to grow anoxically (using liquid paraffin oil on top of the broth) at an initial DO of 0.07 mg L^−1^ or aerobically at an initial DO of 6.78 mg L^−1^. The supernatant was spectrophotometrically assessed (model UV-1800, SHIMADZU EUROPA GmbH) for PO_4_^3−^-P using the molybdenum blue method at 660 nm (absorption peak of phosphomolybdate complex) (Krishnaswamy et al., 2009). The DO levels were regularly measured using a HANNA DO meter (model HI9146-04).

To assess poly-P accumulation, the 24 bacterial isolates were left to grow for 72 h in the abovementioned aerobic or anoxic medium. Next, the bacteria were sonicated and poly-P granules were extracted from the cells. They were quantified spectrophotometrically (model UV-1800, SHIMADZU EUROPA GmbH) at 630 nm by mixing the cell-free poly-P extracts, aqueous toluidine blue (30 mg L^−1^), and 0.2 M acetic acid in a 0.1:1:1 ratio (v/v/v) (Ray and Mukherjee, 2015a). The dry weights of cell masses were also recorded.

To visualize the poly-P granules extracted from the bacterial isolates, 4′, 6 –diamidino-2-phenylindoledihydrochloride (DAPI) staining was conducted followed by fluorescence microscopy (Dewinter OPTIMA-FL Upright Epifluorescence microscope), with excitation at 370 nm and emission at 526 nm (Ray and Mukherjee, 2015b). For comparison, a 2 μg μl^−1^ solution of sodium phosphate glass type 45 (Sigma Aldrich) was visualized under the same conditions.

### 2.11 NGS analysis

The genomic DNA was extracted from CR_R_, M_R_, and HNG_R_ soil samples using NucleoSpin Soil (MACHEREY-NAGEL) kit. A NanoDrop spectrophotometer at 260 and 280 nm was used to assess the quality of the metagenome. A Nextera XT Index Kit (Illumina Inc.) was used to prepare 2 × 300-bp MiSeq libraries. An Illumina MiSeq platform was used to generate FASTQ sequence files for further bioinformatic analyses. The DADA2 pipeline in R 4.2.2 was used to filter and trim the demultiplexed paired-end FASTQ files (Callahan et al., 2016), trimmed at position 20 in both the forward and reverse reads according to the quality profile. Chimeric sequences were removed. An amplicon sequence variant (ASV) sequence table was prepared, followed by taxonomic assignment using “silva_nr99_v138.1” with a minimum bootstrap value of 80. The “ggplot2” package was used to visualize the relative abundance of bacterial taxa. The ampvis2 package was used to visualize the relative abundances of bacterial taxa (Andersen et al., 2018). All the NGS raw data presented in the manuscript were submitted to NCBI. BioProject IDs, BioSample IDs, and SRA IDs (FASTQ files) were granted by NCBI (Supplementary Data).

### 2.12 Statistical analyses and software

Map of study sites was generated through QGIS software (version 3.28.13). All the experiments were carried out with three biological replicates and three technical replicates for each. The mean ± standard error was calculated for all analyses. Analysis of variance (ANOVA) along with Tukey’s honest significant difference (HSD) test were used to determine whether there were significant differences in variables of interest. Values designated with different letters are significantly different at the 5% level according to Tukey’s HSD test (Supplementary Data). Ridgeline plots were generated using the ggplot2 and ggridges packages in R. A heatmap was generated using ggplot2 packages. Bar plots and 3D diagrams were drawn in SigmaPlot 15.0. Dot plots and circular bar plots were constructed using the ggplot2 package in R 4.2.2. Paired dot plots were prepared in GraphPad Prism 9. Scatter plots, scatter plots with 75% confidence ellipses, and Pearson’s correlation analysis on scatter plots were generated using the ggplot2 and ggpubr packages in R 4.2.2.

## 3 Results and discussion

### 3.1 Characterizing narrow ranges of parameters in estuarine mangrove niches in the Indian Sundarbans

**Figure 1:**
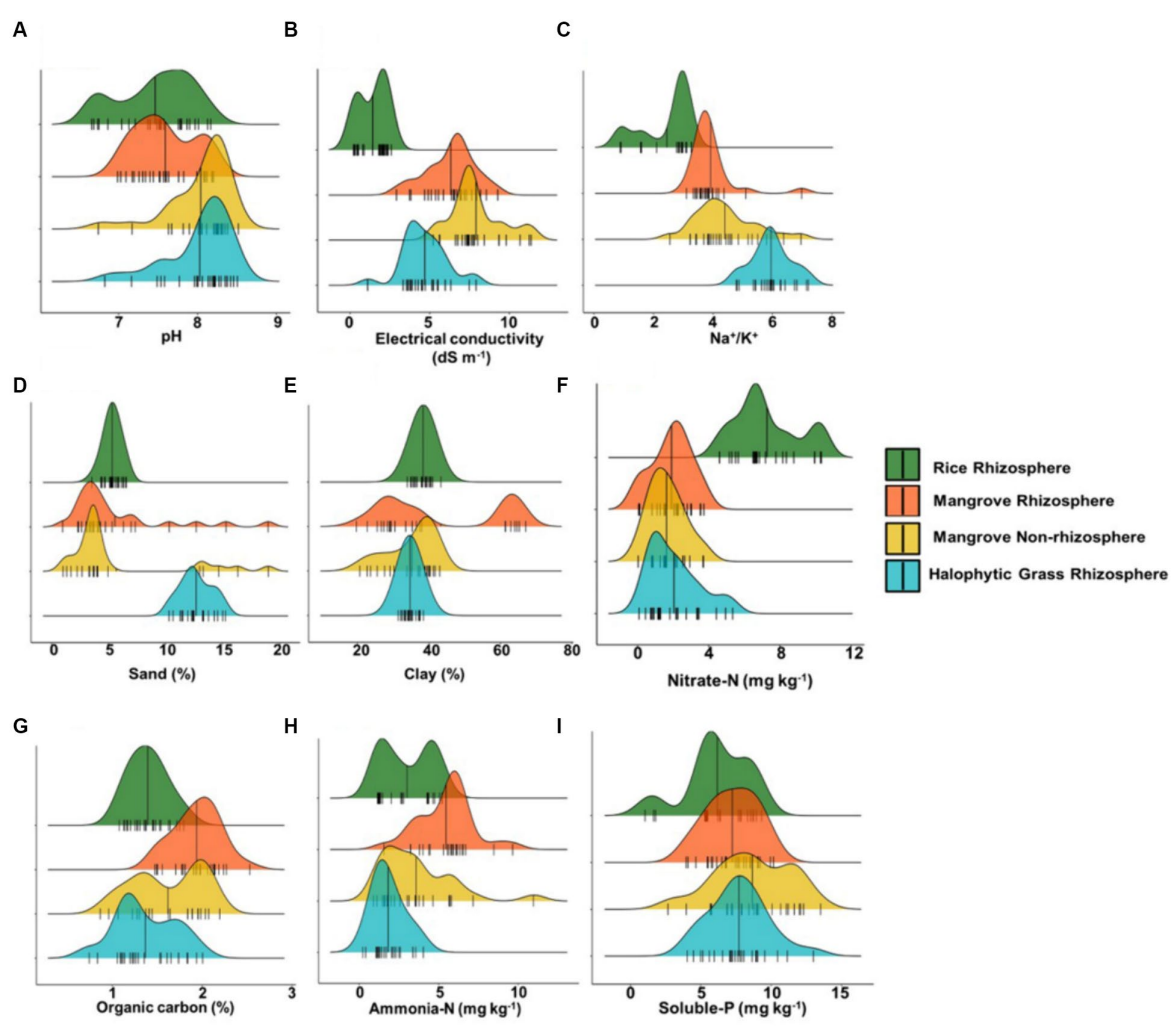
Ridgeline plots representing distribution of different physical and chemical criteria of sediments across Cultivated Rice Rhizosphere, Mangrove Rhizosphere, Mangrove Non-rhizosphere and Halophytic Grass Rhizosphere. **(A)** pH. **(B)** Conductivity. **(C)** Na^+^/K^+^ ratio. **(D)** Sand%. **(E)** Clay%. **(F)** Nitrate-N (mg Kg^−1^). **(G)** Organic carbon%. **(H)** Ammonia-N (mg Kg^−1^). **(I)** Soluble-P (mg Kg^−1^). Here *n* = 25.

Major physical and nutrient parameters were compared across CR_R_, M_R_, M_NR_, and HNG_R_ sediments, and clear trends (within narrow ranges) were detectable (Figure 1; Supplementary Data). While CR_R_ had a broad pH distribution of ~6–8, M_NR_ and HNG_R_ had slightly alkaline pH, with pH peaking mostly >8 (Figure 1A). In contrast, the pH of M_R_ was intermediate and restricted, at 7–8 (near neutral) (Figure 1A). Alkaline pH drives most of the soluble nutrients (such as Fe and P) to convert to insoluble forms in mangrove sediments, causing nutrient limitations (especially for FLNF) (Alongi, 2010; Reef et al., 2010). A regression tree analysis of conserved and reforested mangrove sites revealed pH was a major controller of BNF, which may be influenced by tidal water temperature, DO, and organic acids released from detritus (Vovides et al., 2011). BNF has been reported to peak at pH 6.49; above this pH, nitrogenase activity was suppressed, with some enhancement at >28.6°C (Vovides et al., 2011). However, with increasing soil pH, the α-diversity of the resident diazotrophic community increased linearly in grassland soil of alpine meadows of the high-altitude Qinghai-Tibet Plateau (Wang et al., 2017). In this grassland soil, the relative abundances of three major diazotrophs (FLNF *Azospirillum* sp., FLNF *Bradyrhizobium* sp., and symbiotic diazotroph *Mesorhizobium* sp.) increased across a pH gradient of 7–8, revealing that pH fluctuations may influence the abundances of at least these major resident diazotrophic species (Wang et al., 2017; Smercina et al., 2019). At pH 7–8 compared to 5–7, there was more clustering of diazotroph taxa (Wang et al., 2017).

Electrical conductivity (EC) depends on the total labile ionic species and is related to salinity. It strongly fluctuated within 7–9 dS m^−1^ for M_R_ and M_NR_, sometimes up to 11 dS m^−1^ for M_NR_ (Figure 1B), depending on the duration and frequency of submergence in hypersaline (~18–45 dS m^−1^) (Supplementary Data) estuarine tidal water (Chowdhury et al., 2019). HNG_R_ exhibited a somewhat lower range of EC (~3–6 dS m^−1^) (Figure 1B). As expected, the range of EC for salt-sensitive CR_R_ was 0.2–2 dS m^−1^ (Figure 1B). Increasing salinity in mangrove niches limits both P and N availability, as previously observed by our group (Chowdhury et al., 2019). Microbial hydrolyzing enzyme activities responsible for P and N release in mangrove niches also decreased with increasing salinity (Batra and Manna, 1997). In degraded mangroves, salinity apparently controlled the activity of diazotrophic bacteria. At lower salinity (<47.6%), nitrogenase activity increased, though increased to some extent at low pH (Vovides et al., 2011).

The sediment cores’ temperature profile across different study sites (Supplementary Data; Supplementary Information) also revealed interesting trends. It demonstrated ~ ± 0.5–1°C variation between pre-monsoon and monsoon temperatures at 15 and 30 cm depths within broadly ~29–33°C range (Supplementary Data). In contrast, post-monsoon sediment temperatures showed a plummet of ~7–10°C from pre-monsoon and monsoon temperatures across study sites at both depths. Soil temperature is supposed to vary both temporally (seasonally, even from month to month) and spatially across the sites. Sediment temperatures reported earlier from Indian Sundarbans from a different set of study sites were demonstrated to be 17.8 ± 6.6°C at pre-monsoon, 24.4 ± 2.9°C at monsoon and 13.0 ± 5.7°C at post-monsoon across 0–60 cm vertical depth (Ray et al., 2014). Although these records seemed less comparable with our data, maybe because of representing crude averages across a broad range, a high diazotroph bacterial abundance in sediments was reported in October and April (during the post and pre-monsoon sediment temperature regime) while the lowest abundance of the same was observed in July (at monsoon sediment temperature), in the same study (Ray et al., 2014). N_2_ fixation was experimentally demonstrated to be temperature-dependent in Mediterranean macrophyte meadow for both bare and vegetated sediments (Garcias-Bonet et al., 2019), where the optimal temperature for BNF was shown to be 31°C with a sharp decrease at 33°C. In seagrass rhizosphere sediment N_2_ fixation rates in vegetated and bare sediments were optimal at 28.5°C and decreased further at both lower and higher temperatures (Garcias-Bonet et al., 2018). Similarly, N_2_ fixation evaluated via acetylene reduction assay (ARA) from biological soil crusts of different successional stages showed a steady increase up to 15–20°C, then started plateauing and declined at 30–35°C (Zhou et al., 2016). The saturation effect of BNF at higher temperatures was attributed to the inability of the diazotrophs to grow at higher temperatures.

The surface hydrological profile across the study sites (Supplementary Data; Supplementary Information) was also recorded. It demonstrated a sharp fall in EC during monsoon, the highest drop in temperature post-monsoon, the pH showing little variation across seasons (ranging ~7.3–8.3), DO ranging between ~6.2–8.7 ppm seasonally and the turbidity being the highest (198.6 NTU in August–September) during monsoon due to obvious sediment transport from intertidal mudflats. Earlier research (Dutta et al., 2017), conducted at similar study sites of the western part of the Indian Sundarbans (along the Saptamukhi riverine estuary) corroborated our observations that seasonal pH difference was not significant (~8.1–8.17) with observed DO level in estuarine surface and sub-surface waters being quite high, indicating a well-mixed oxygen-rich water column. A similar decline in salinity at monsoon and temperature at post-monsoon was observed also with pre-monsoon and monsoon water temperatures ranging between ~28–30°C (Dutta et al., 2017). Another study from the Jharkhali estuary (a part of the Hooghly-Matla estuary) in the eastern part of the Indian Sundarbans (Chaudhuri et al., 2012), validated the same hydrological criteria, with the lowest water surface temperature in post-monsoon (21.5°C in January), pH least variable (~8–8.15), salinity being the lowest at monsoon (12.6 PSU in October), DO ranging between ~6.5–9.8 ppm, with maximum turbidity observed at monsoon (125 NTU in October). Mangrove study sites in Mexico observed the influence of pore-water salinity and pH on sediment nitrogen fixation (Vovides et al., 2011). In this arid region during the summer months (July–September), at an average temperature of 30.5°C, with 90% rainfall, and with highest surface and subsurface freshwater inputs, a higher amount of organic acids released from detritus, altogether lowered the pH and salinity in sediment as well as in pore-water, and nitrogen fixation rates via culturable heterotrophic diazotrophs was found to be greater during this time (Vovides et al., 2011). The research involving sea surface water warming effect on N_2_ fixation rates of marine macrophytes in the Mediterranean Sea (Garcias-Bonet et al., 2019) demonstrated that average summer sea surface temperature ranging between 22.92°C and 29.08°C (recorded during 2013–2017) was correlated with the observed optimum temperature of N_2_ fixation at 31°C, in vegetated macrophyte meadows, and was concluded that the forecasted warming might increase the N_2_ fixation rate causing higher productivity in Mediterranean macrophytes.

The extremely high Na^+^/K^+^ ratios of 5–7 in HNG_R_ were indicative of lower intertidal locations that were diurnally inundated with hypersaline (~18–45 dS m^−1^) (Chowdhury et al., 2019; Supplementary Data) tidal water (Figure 1C). For M_R_ and M_NR_, the Na^+^/K^+^ ratio peaked at ~3–5, and for CR_R_, the ratio was ~2–3 (Figure 1C). Exchangeable Na^+^ was clearly predominant, as an inherent component of estuarine mangrove sediments.

HNG_R_ sediments were 11–12% sand, while CR_R_, M_R_, and M_NR_ sediments had a rather low sand content of 3–6% (Figure 1D). CR_R_ and HNG_R_ sediments had similar clay contents of ~35–40% and ~ 32–35%, respectively (Figure 1E). M_NR_ sediments had ~20–40% clay content while M_R_ sediments had a wide range (~20–65% with major peaks at ~20–40% and ~ 60–65%) (Figure 1E). Increased clay content with adequate organic C availability is presumed to raise the probability of FLNF colonization (with increased nitrogenase activity) at microaerobic and anaerobic microsites formed by clay particle and organic C aggregation (Gupta and Roper, 2010; Smercina et al., 2019). Both sand and clay percentages have a significant predictive power for BNF (by both symbiotic diazotrophs and FLNF) (Davies-Barnard and Friedlingstein, 2020). However while clay positively correlated with FLNF activity, sandy soils were not preferred for the same (Gupta and Roper, 2010).

Nitrate-N was notably low (1–2 mg kg^−1^) in M_R_, M_NR_, and HNG_R_, while it was 4–10 mg kg^−1^ in CR_R_ sediment sampled after rice harvesting (Figure 1F). In contrast, M_R_ had higher ammonia-N (generally being ~5–6 mg kg^−1^) while CR_R_, M_NR_, and HNG_R_ had 1–4 mg kg^−1^ (Figure 1G). Mangrove niches are deficient in nitrate-N due to higher net denitrification in the sediments (Alongi et al., 2002; Alongi, 2013, 2020); it has repeatedly been reported that ammonia-N is the primary N source in mangrove habitats, especially in M_R_ (Alongi et al., 2002; Reef et al., 2010; Alongi, 2013, 2020).

In the M_R_, M_NR_, and HNG_R_ sediments, the organic C content was high (~1–2%) because of its integral association with carbohydrate-rich organic matter resulting from detritus/litter in mangrove-related sediments (Figure 1H). The organic C content in CR_R_ (~1–1.5%) was comparable (Figure 1H). FLNF have a positive association with diverse and complex organic C dissolved in the soil, while symbiotic diazotrophs receive simple C nutrients directly from their host plants (Smercina et al., 2019). C sources in rhizospheres are invaluable to heterotrophic FLNF in rhizospheres as they allow ATP to be obtained through respiration for effective BNF (Dynarski and Houlton, 2018). A statistical model indicated that soil organic C weakly predicted BNF (Davies-Barnard and Friedlingstein, 2020).

All four sediment types had a similar distribution of soluble P (~4–12 mg kg^−1^, mostly ~6–9 mg kg^−1^) (Figure 1I). Soluble P (rather than N) availability in rhizospheric niches is a well-known driver of FLNF (Smercina et al., 2019). P limitation in niches with FLNF is more common in tropical areas. Multivariate linear regression indicated that P supplementation increased BNF (as high as 25-fold) by increasing niche net primary productivity and substrate C/N ratio (Dynarski and Houlton, 2018). BNF and net primary productivity were both negatively associated with substrate N concentrations; BNF responses to N or N + P supplementation were negatively associated with the substrate N/P ratio (Dynarski and Houlton, 2018). In natural soil environments, soluble P availability depends primarily on microbial P mobilization via either organic-P mineralization (by releasing phosphatases) or bound inorganic-P solubilization (by releasing protons in the form of organic acids for acidification) (Widdig et al., 2019). N supplementation reduced the relative abundance of PSB, changed the PSB compositional profile, and augmented phosphatase activity, whereas P supplementation had no effect. Thus, N supplementation can shift the soil P mobilization routes from P solubilization to P mineralization (Widdig et al., 2019). In N-deficient mangrove niches, PSB may have an indispensable role in soluble P availability. Our comprehensive analysis of mangrove rhizospheric/non-rhizospheric niches demonstrated that, despite having greater clay% and high soil organic C (two favorable criteria for heterotrophic FLNF), the niches are quite challenging and stringent for heterotrophic FLNF, primarily because of the alkaline pH trend, higher temperature at pre-monsoon and monsoon, high salinity, and high Na^+^/K^+^ ratio, with available P and N deficiency in mangrove habitats, as observed in this study. BNF in mangrove niches was found to be inversely proportional to the available N/P ratio (Howarth and Marino, 1988; Vovides et al., 2011). High N and low P in the niche suppressed BNF, while low N and high P enhanced BNF. Hence, the estuarine mangrove littoral environment in the Indian Sundarbans, with its high pH, high temperature, high salinity, high Na^+^/K^+^ ratio, and unfavorable N/P ratio, represents a hostile narrow niche for BNF (Figure 1).

### 3.2 Heterotrophic nutrient cyclers’ abundances across narrow mangrove niche parameters

**Figure 2:**
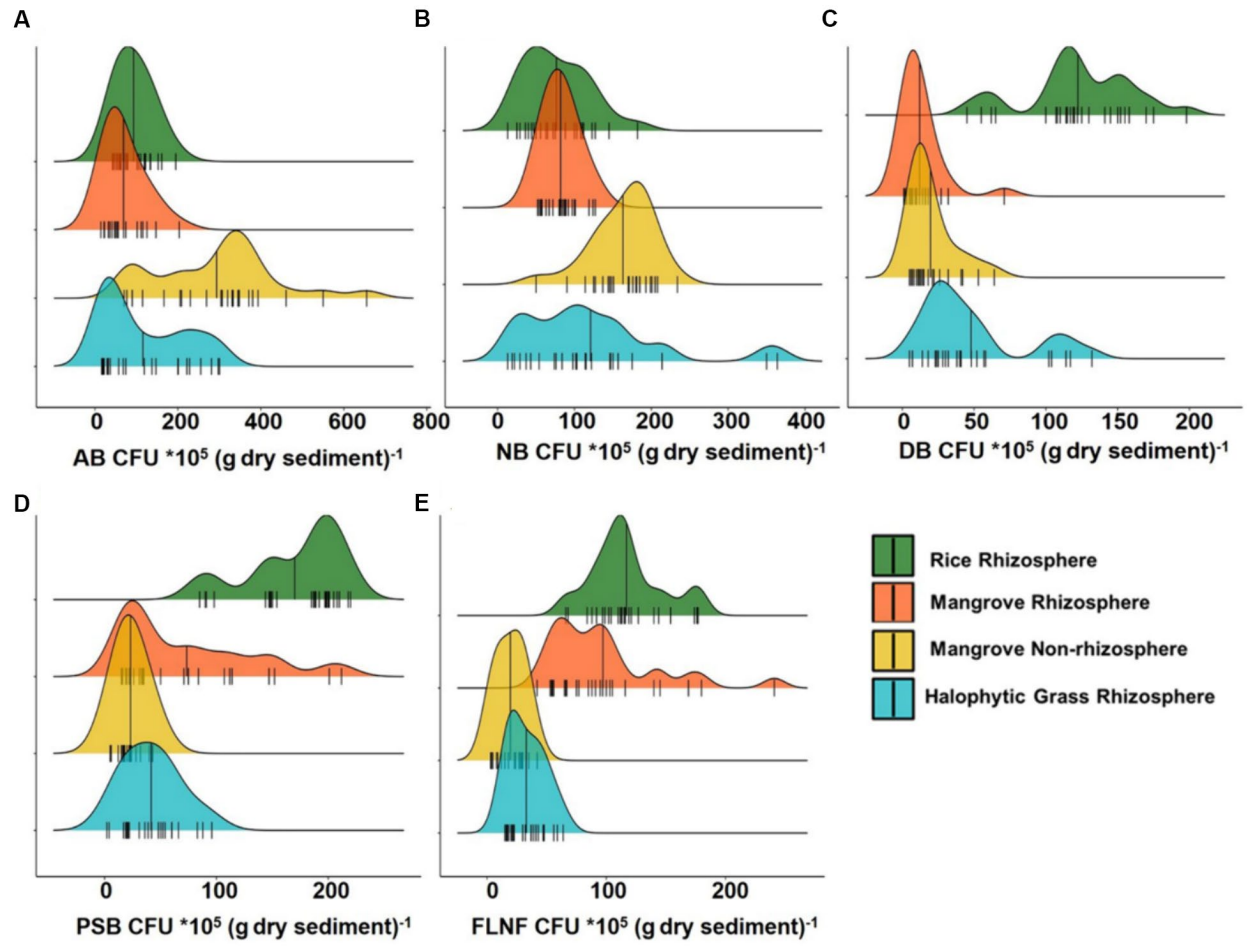
Ridgeline plots showing distribution analysis of Colony Forming Unit (CFU) of major nutrient cycling bacteria from Cultivated Rice Rhizosphere, Mangrove Rhizosphere, Mangrove Non-rhizosphere and Halophytic Grass Rhizosphere. **(A)** CFU of Ammonifying Bacteria (AB). **(B)** CFU of Nitrifying Bacteria (NB). **(C)** CFU of Denitrifying Bacteria (DB). **(D)** CFU of Phosphate Solubilizing Bacteria (PSB). **(E)** CFU of Free Living Nitrogen Fixing Bacteria (FLNF). Here *n* = 25.

Interestingly, the CFU counts of various nutrient cyclers (AB, NB, DB, PSB, and FLNF) from the four sediment types on various differential growth selection media (Figure 2; Supplementary Data) did not accord wholly with the mangrove niche nutrient parameters depicted in Figure 1.

AB had the highest CFU count among all the decomposer types assessed. M_NR_ had the widest CFU range for AB (Figure 2A), but ammonia-N was highest in M_R_ (Figure 1H). This might be because M_R_ may be an ideal niche for FLNF, based on the CFU count (Figure 2E). Combining the AB and FLNF activity in M_R_ may explain the higher ammonia-N (Figure 1H), corroborating the previous findings of a net high ammonification potential of mangrove niches (Alongi et al., 2002; Reef et al., 2010; Alongi, 2013, 2020).

Similarly, the low CFU count of NB (Figure 2B), particularly in CR_R_, did not accord with the high nitrate-N in CR_R_ (Figure 1F). This might relate to inorganic N-fertilizer addition, or any other anthropogenic inputs of inorganic-N, to the CR_R_ niche. The CFU count of DB peaked in CR_R_, (Figure 2C), which may be associated with the high nitrate-N in CR_R_ (Figure 1F). The hardly detectable nitrate-N in M_R_, M_NR_, and HNG_R_ (Figure 1F) may be related to the lower CFU counts of NB (Figure 2B) and DB (Figure 2C) in these niches, concurring with the higher net denitrification potential of mangrove niches (Alongi et al., 2002; Reef et al., 2010; Alongi, 2013, 2020).

The CFU count of PSB was higher in CR_R_ than in the other three sediment types (Figure 2D) whereas soluble P distribution was comparable in all sediment types (Figure 1I). It can be assumed that PSB in the three sediment types with high salinity (M_R_, M_NR_, and HNG_R_) exhibited enhanced P solubilization efficiency in order to compensate for the low CFU count of PSB, increasing soluble P-release. This intriguing observation validated previous findings that the P solubilization efficiency of PSB increased with increasing salinity (Srinivasan et al., 2012).

CFU counts of FLNF were more widely distributed in M_R_ and CR_R_ compared to M_NR_ and HNG_R_ (Figure 2E), indicating that the former two are more appropriate for FLNF colonization. Interestingly, the CFU abundance distribution width overlap for both PSB and FLNF for all mangrove-related rhizosphere types (M_R_, M_NR_, and HNG_R_) was significantly evident (Figures 2D,E) except for the CR_R_. This indicates the possible co-occurrence/positive correlation of PSB and FLNF in estuarine mangrove niches (Figure 2).

### 3.3 Bacterial abundances in mangrove niches: cultivation-independent vs. cultivation-dependent analyses

**Figure 3:**
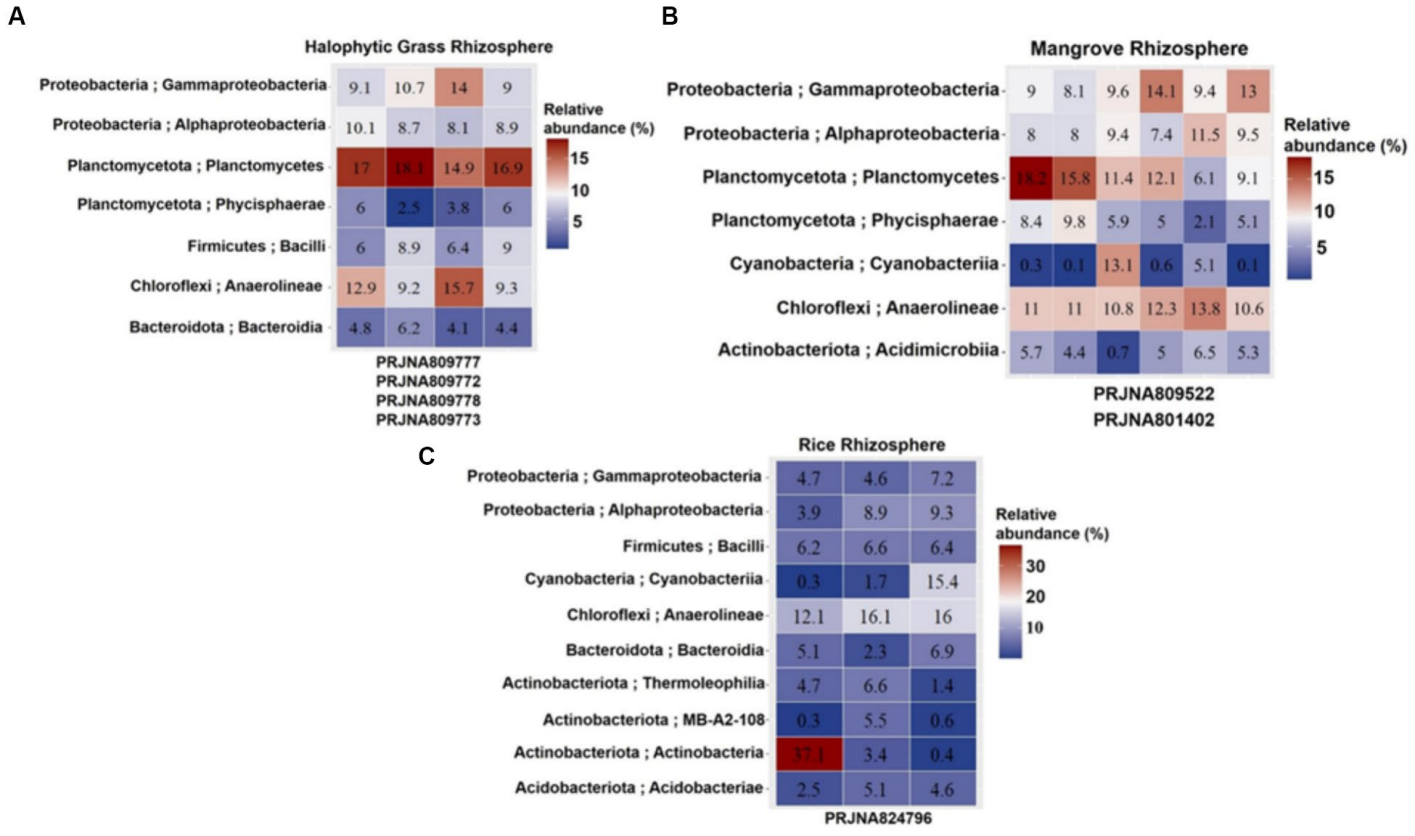
**(A)** Halophytic Grass Rhizosphere. **(B)** Mangrove Rhizosphere. **(C)** Cultivated Rice Rhizosphere. Heatmap displaying top 7–10 bacterial classes (Y-axis) defined at 3 different estuarine mangrove-related rhizospheres from Indian Sundarbans (labeled in columns). The relative abundance is shown in a > 5% scale with three color variants. Read higher abundances in red gradient and lower abundances in blue gradient. The heatmap was built on different Biosample reads under different NCBI Bioprojects involving mangrove-related rhizosphere types (X-axis).

Cultivation-independent analyses of 16S rRNA abundances (based on NGS of the 16S rRNA V3–V4 region) in the HNG_R_, M_R_ and CR_R_ were used to assess bacterial diversity and abundances (>5% in Figure 3 and < 5% in Supplementary Figure S2). V3-V4 primer pair-based analyses generated 99,523–259,157 raw reads and 3,065–45,119 filtered reads analyzed across the SRA data under each bioproject (Supplementary Data; Supplementary Table S1) representing the actual bacterial diversity prevailing in the respective rhizospheres. Anaerolineae (~9.2–15.7%), Planctomycetes (~6.1–18.2%), Gammaproteobacteria (~9–14.1%), Alphaproteobacteria (~8–11.5%), and Phycisphaerae (a class belonging to the phylum Planctomycetota; 2.1–9.8%) had high relative abundances in both HNG_R_ (PRJNA809777, PRJNA809772, PRJNA809778, and PRJNA809773) and M_R_ (PRJNA801402, PRJNA809522, PRJNA809522, PRJNA801402, PRJNA801402, and PRJNA801402) (Figure 3; Supplementary Figure S2). There was a high relative abundance of Anaerolineae (~12.1–16.1%) and lower relative abundances of Planctomycetes (~1.4–4.4%), Gammaproteobacteria (~4.6–7.2%), Alphaproteobacteria (~3.9–9.3%), and Phycisphaerae (0.3–2%) in CR_R_ (PRJNA824796, PRJNA824796, and PRJNA824796). Bacilli was moderately high in HNG_R_ (~6–9%) and CR_R_ (~6.2–6.6%), but relatively low in M_R_ (~0.8–3.5%). Actinobacteria was relatively low in HNG_R_ (~0.5–1.8%) and M_R_ (~0.2–2.2%), and in CR_R_ (~0.4–3.4%), except in one abnormal biosample (~37.1%). Cyanobacteria had an abundance of 13.1% in one M_R_ biosample and 15.4% in one CR_R_ biosample, with the remainder ranging within 0.2–5.1%. Other classes had very low relative abundances for all biosamples (Supplementary Figure S2).

**Figure 4:**
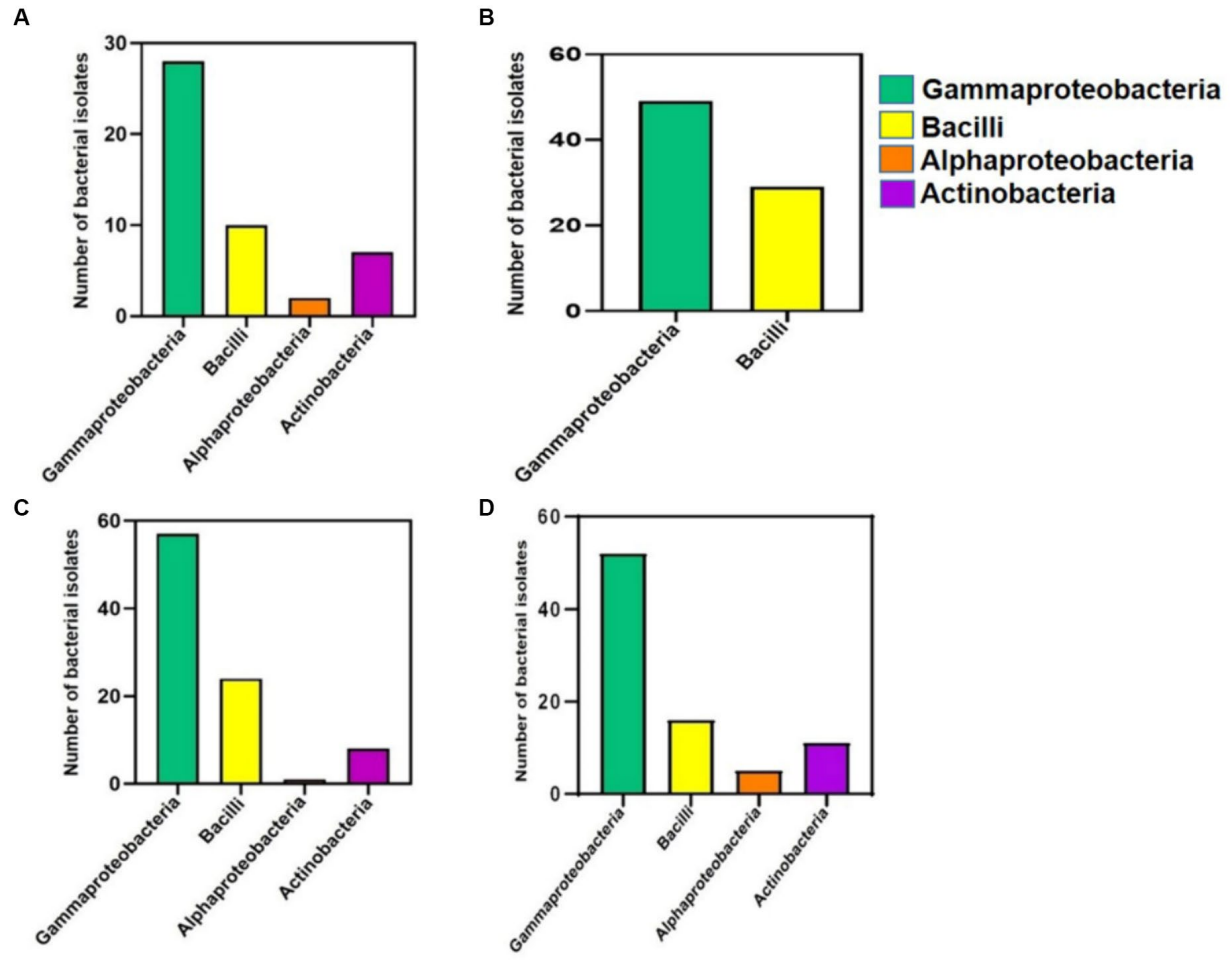
Bar diagrams displaying the no. of cultivable bacterial isolates developed and accessioned in NCBI, depicted class-wise (Y-axis) for 4 different mangrove-related rhizospheres/root sources (X-axis). **(A)** Cultivated Rice Rhizosphere. **(B)** Mangrove Root endophytes. **(C)** Halophytic Grass Rhizosphere. **(D)** Mangrove Rhizosphere.

When the class abundances from the cultivation-independent analysis observed, were compared to the classes of the 299 accessions from the cultivation-dependent analysis (Figure 4), which were obtained from CR_R_ (47 accessions, accession numbers MT145945–MT145991), MRP_E_ (78 accessions, accession numbers MT421976–MT422053), HNG_R_ (90 accessions, accession numbers MT145456–MT145545), and M_R_ (84 accessions, accession numbers MH910698–MH910745, MH910747–MH910768, MH910775, MH910778, and MH910785–MH910796), the results were found to be highly similar, validating each other.

In the cultivation-independent analysis, the class Anaerolineae, phylum Planctomycetota, class Gammaproteobacteria, and class Alphaproteobacteria were the most abundant bacteria in both HNG_R_ and M_R_. The former two are rarely reported to be culturable, while many of the latter two are highly culturable. In the cultivation-dependent analysis, there were many Gammaproteobacteria isolates in M_R_ and HNG_R_ (61.9% [52 isolates] and 63.3% [57 isolates], respectively) but fewer Alphaproteobacteria isolates (5.9 and 1.1%, respectively) (Figure 4). Due to the high culturability of Gammaproteobacteria, there were many Gammaproteobacteria isolates in CR_R_ and MRP_E_ (59.5% [28 isolates] and 62.8% [49 isolates], respectively), but very few Alphaproteobacteria isolates (4.2% [2 isolates] and 0%, respectively) (Figure 4).

In the cultivation-independent analysis, Bacilli (another frequently culturable class) had moderately high relative abundances in all groups and, in the cultivation-dependent analysis, 21% (10 isolates), 37% (29 isolates), 26.6% (24 isolates), and 19% (16 isolates) were cultured from CR_R_, MRP_E_, HNG_R_, and M_R_, respectively (Figure 4). Despite the low relative abundances, there were cultured Actinobacteria isolates from CR_R_ (14.9%, 4 isolates), HNG_R_ (8.8%, 8 isolates), and M_R_ (13%, 11 isolates), but not from MRP_E_ (Figure 4).

In summary, the cultivation-independent vs. cultivation-dependent analyses unequivocally established high relative abundances of Gammaproteobacteria and Bacilli (both of which have high culturability) in the estuarine mangrove niches. The high culturability and high abundance of Gammaproteobacteria were related, very much in agreement with the high culturability of marine Proteobacteria genera reported by previous researchers (Ye et al., 2016; Zhang W. et al., 2019; Zhang J. et al., 2019).

The findings of previous cultivation-independent analyses of samples from estuarine/riverine mangrove ecosystems in the Indian Sundarbans in the last two decades (Ghosh et al., 2010; Basak et al., 2015a,b, 2016; Chakraborty et al., 2015; Ghosh and Bhadury, 2018; Biswas and Mukherjee, 2019; Dhal et al., 2020) are similar to our observations in many respects. There were eight major phyla in mangrove sediments from the Indian Sundarbans, comprising Proteobacteria, (alpha, beta, gamma, and delta), Flexibacteria (Cytopahga-Flexibacteria-Bacteroides [CFB] group), Actinobacteria, Acidobacteria, Chloroflexi, Firmicutes, Planctomycetes, and Gemmatimonadetes, and the class Gammaproteobacteria mostly dominated the sequenced library clones (Ghosh et al., 2010). Many of the Gammaproteobacteria sequences resembled sulfur oxidizers while the Deltaproteobacteria sequences showed major similarities to sulfur- and sulfate-reducing bacteria (Ghosh et al., 2010). In spatiotemporal analyses of 16S rRNA pyrosequencing results based on mangrove sediments, Proteobacteria was found to be abundant, including Deltaproteobacteria, Alphaproteobacteria, and Gammaproteobacteria (Basak et al., 2015a). An analysis of the bacterial community profile of mangrove sediments from variable depths of the Indian Sundarbans (using 16S rRNAamplicon sequencing) further reported that Proteobacteria and Firmicutes were the most abundant bacterial phyla, with abundance varying by depth (Basak et al., 2015b). A similar analysis of bacterial diversity at Indian Sundarbans sites exposed to anthropogenic activities revealed associations between environmental pollution and the diversity of bacterial isolates with genes for hydrocarbon degradation and heavy metal tolerance (based on 16S rRNA libraries) (Chakraborty et al., 2015). An analysis of sediments from pristine mangroves of Dhulibhashani in the Indian Sundarbans (based on 16S rRNA amplicon sequencing data) showed that Proteobacteria was the dominant phylum among the 44 phyla identified, and there were high abundances of Bacteroidetes, Acidobacteria, Firmicutes, Actinobacteria, Nitrospirae, Cyanobacteria, Planctomycetes, and Fusobacteria (Basak et al., 2016). A study of surface waters near Sagar Islands in the Sundarbans mangrove environment (using 16S rRNA clone library and Illumina MiSeq approaches) reported that Proteobacteria of two major classes (Gammaproteobacteria and Alphaproteobacteria) exhibited similar dominance in monsoon vs. post-monsoon seasons (Ghosh and Bhadury, 2018). The Proteobacteria-dominated sites were associated with uncultured Planctomycetes and Chloroflexi (for N cycling) (Ghosh and Bhadury, 2018). Sphingomonadales, Chromatiales, Alteromonadales, Oceanospirillales, and Bacteroidetes sequence richness (which contribute to coastal C cycling) was also identified (Ghosh and Bhadury, 2018). The large increases in the abundances of Firmicutes and *Desulfovibrio* in the topmost water layers in the monsoon seasons indicated the resuspension of sediment-inhabiting bacteria in the topmost water layers (Ghosh and Bhadury, 2018). These cultivation-independent analyses indicated that there are seasonal fluctuations, such as low Proteobacteria abundances in surface sediments in monsoon seasons and higher abundances in subsurface sediments in post-monsoon seasons. In another study of Sundarbans mangroves, Gammaproteobacteria and Deltaproteobacteria were the two most abundant classes, with the latter related to anaerobic sediments enriched with sulfate-reducing bacteria (Biswas and Mukherjee, 2019). An analysis of coastal waters of Matla and Thakuran rivers at Maipith in the Indian Sundarbans (involving 16S rRNA amplicon sequencing) indicated the dominance of halophilic marine bacteria from the family Flavobacteriaceae (Dhal et al., 2020). In eutrophic open marine water zones, the families Oceanospirillaceae and Spongiibacteraceae (encompassing bacteria known for marine hydrocarbon degradation) were found (Dhal et al., 2020). The family Rhodobacteraceae and domain Archaea were also dominant in both riverine aquatic environments (Dhal et al., 2020).

Although only 0.1–1.0% of environmental bacteria are culturable (Zeyaullah et al., 2009), there are many studies involving culturing bacteria from Sundarbans mangrove sediments (Ramanathan et al., 2008; Barua et al., 2012; Das et al., 2012; Dhal et al., 2020; Pallavi et al., 2023). Three estuarine sites in the Indian Sundarbans (Canning, Jharkhali, and Pakhiraloy) exhibited high CFU counts of PSB, FLNF, and cellulose-degrading bacteria (Ramanathan et al., 2008). In Sundarbans mangrove sediments from three pristine mangrove sites (Patharpratima, Lothian Island, and non-rhizospheric zones of the Saptamukhi estuarine mouth), the dominant culturable nutrient cyclers were cellulose-degrading bacteria, sulfate-reducing bacteria, NB, PSB, and FLNF, which exhibited seasonal and spatial variations (Das et al., 2012). Two salt-tolerant diazotrophic species (*Agrobacterium* SUND_BDU1 and *Bacillus* sp. SUND LM2, Can4, and Can6) were isolated from Sundarbans mangrove sediments and applied to agricultural fields to substitute chemical fertilizers (Barua et al., 2012). Of 156 bacterial isolates from Sundarbans mangrove sediments, 20 had high salt tolerance and four genera/species (*Arthrobacter* spp., *Pseudomonas plecoglossicida*, *Kocuria rosea*, and *Bacillus* spp.) exhibited P and Zn solubilization, indole acetic acid production, and siderophore and ammonia release (Pallavi et al., 2023). These strains were evaluated for plant growth promotion after application on pea plants growing under high salinity (Pallavi et al., 2023). However, none of these cultivation-dependent studies was validated based on cultivation-independent bacterial abundances at the same mangrove sites.

### 3.4 Comparison of four BNF-facilitating functions of FLNFM- vs. IOM-selected isolates

High cultivability and a large no. of FNFLM-selected isolates (142) comparable with a large no. of IOM-selected ones (93) among the 235 pure isolates (comprising 190 accessioned and 45 non-accessioned isolates) developed in this study and their common high P-solubilization rate (60–127 μg mL^−1^) and high siderophore synthesis ability (40–91%) in most of the isolates observed (Supplementary Data) was found to be quite intriguing and led us to undertake the subsequent comparative analyses. One plausible justification for linking FNFLM and IOM-selected isolates via siderophore production was that nitrogenase being an iron-intensive enzyme complex, having both Fe and Mo-Fe protein and Fe-Mo cofactor components (Boyd et al., 2015), the FLNF as well as symbiotic diazotrophs, acquires the oxidized form of iron Fe^3+^, produced by IOBs, via an intact siderophore system that makes iron bioavailable (Fe^3+^, usually an insoluble form at physiological pH) (Neilands, 1995). On the other hand, the P-solubilization trait of FLNF increases the chance of adequate availability of soluble P especially in P-deficient mangrove habitats (Alongi, 2018). Therefore, under laboratory conditions, 142 FLNFM-selected isolates and 93 IOM-selected isolates were evaluated further for four BNF-facilitating functions (log_10_CFU on FLNFM, P solubilization [P release in μg mL^−1^], siderophore production, and time to iron oxidation [number of days required for iron oxide precipitate to be visualized]) (Supplementary Data; Supplementary Information).

**Figure 5:**
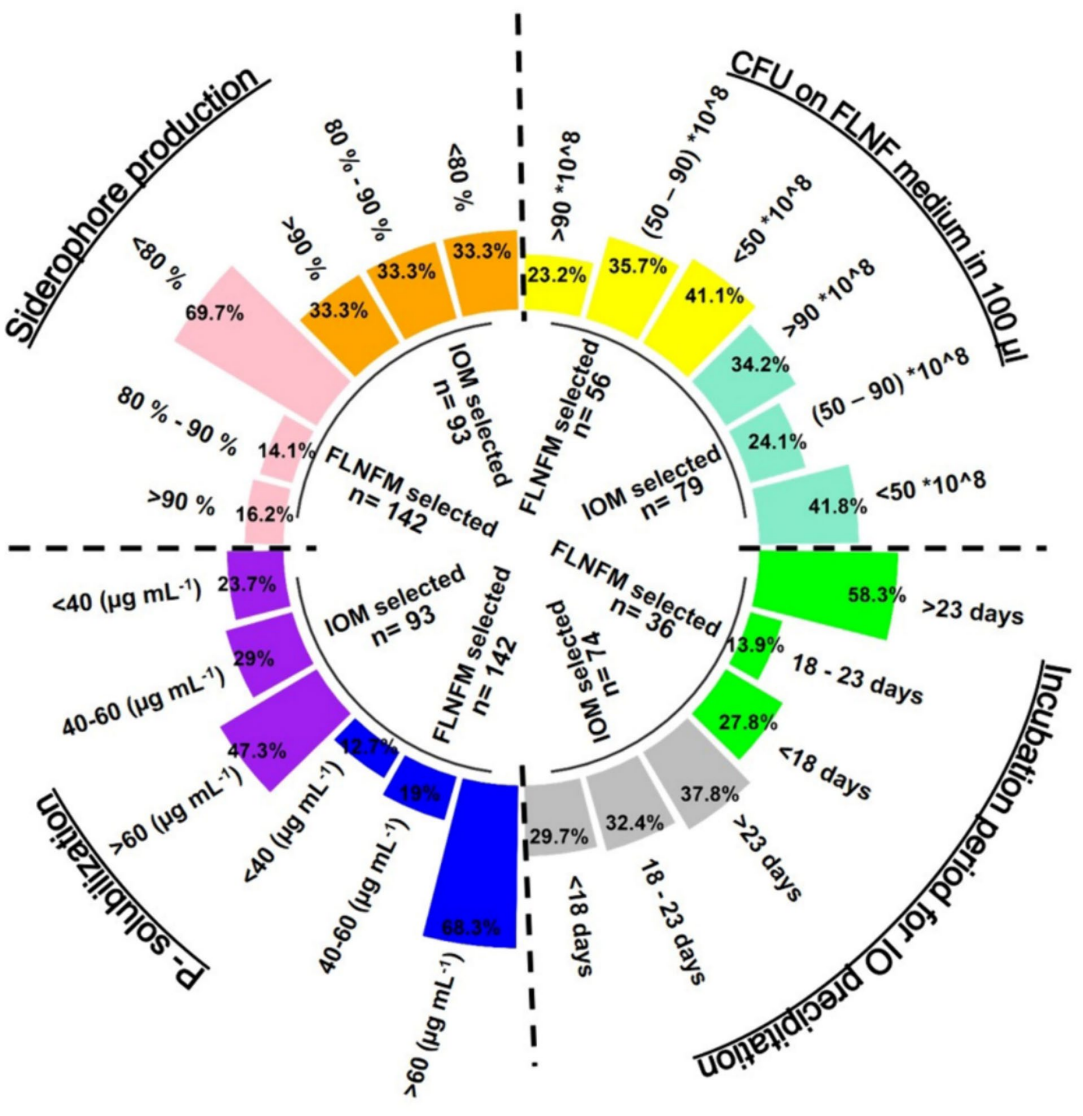
Circular grouped bar diagram representing percentage proportion of stringent (minimal) Iron Oxidation Medium (IOM) selected and Free Living Nitrogen Fixing Medium (FLNFM) selected bacterial isolates performing P-solubilization (<40, 40–60, >60, μg mL^−1^), siderophore production (<80%, 80–90, >90%), Colony Forming Unit (CFU) counts (×10^8^) 100 μL^−1^ on FLNF medium (<50, 50–90, >90) and Incubation period for Iron Oxide (IO) precipitation (<18, 18–22, >23 in days). The dotted lines are the 4 segments of each parameter.

FLNF was selected by repeated streaking and recording of the log_10_CFU on FLNFM and acetylene reduction assays for the function of nitrogenase. Heterotrophic IOBs were selected on a minimal C-supplemented medium with ferrous salt (at a constant sub-neutral pH of 6.0) to observe biological iron oxidation. Efficient IOB-formed iron oxide precipitates rapidly, while less efficient IOB took longer to form visible iron oxide precipitates in the medium. Interestingly, most of the IOBs were found to flourish on FLNFM. This led us to investigate whether the abovementioned four major BNF-facilitating functions were also common for all FLNFM- and IOM-selected isolates in this study (Figure 5). We found that all four functions were present for many of the 235 isolates.

Of the 142 FLNFM-selected isolates, 68.3% were good P-solubilizers (P-release >60 μg mL^−1^), while of the 93 IOM-selected isolates, only 47.3% were good P-solubilizers (Figure 5). Regarding siderophore production, the FLNFM-selected isolates were less efficient (69.7% had <80%) than the IOM-selected isolates (33.3% had >90, 33.3% had 80–90, and 33.3% had <80%) (Figure 5). Similarly, 29.7, 32.4, and 37.8% of 74 IOM-selected isolates took <18, 18–23, and > 23 days, respectively, to form iron oxide precipitate, whereas 58.3% of the 36 FLNFM-selected heterotrophs took >23 days (Figure 5). Intriguingly, the IOM-selected isolates had higher log_10_CFU on FLNFM, with 34.2% of the 79 IOM-selected isolates and only 23.2% of the 56 FLNFM-selected isolates having a value >90 × 10^8^ (Figure 5). Based on this analysis, IOM-selected heterotrophs were better at the four BNF-facilitating functions, at least under laboratory conditions.

**Figure 6:**
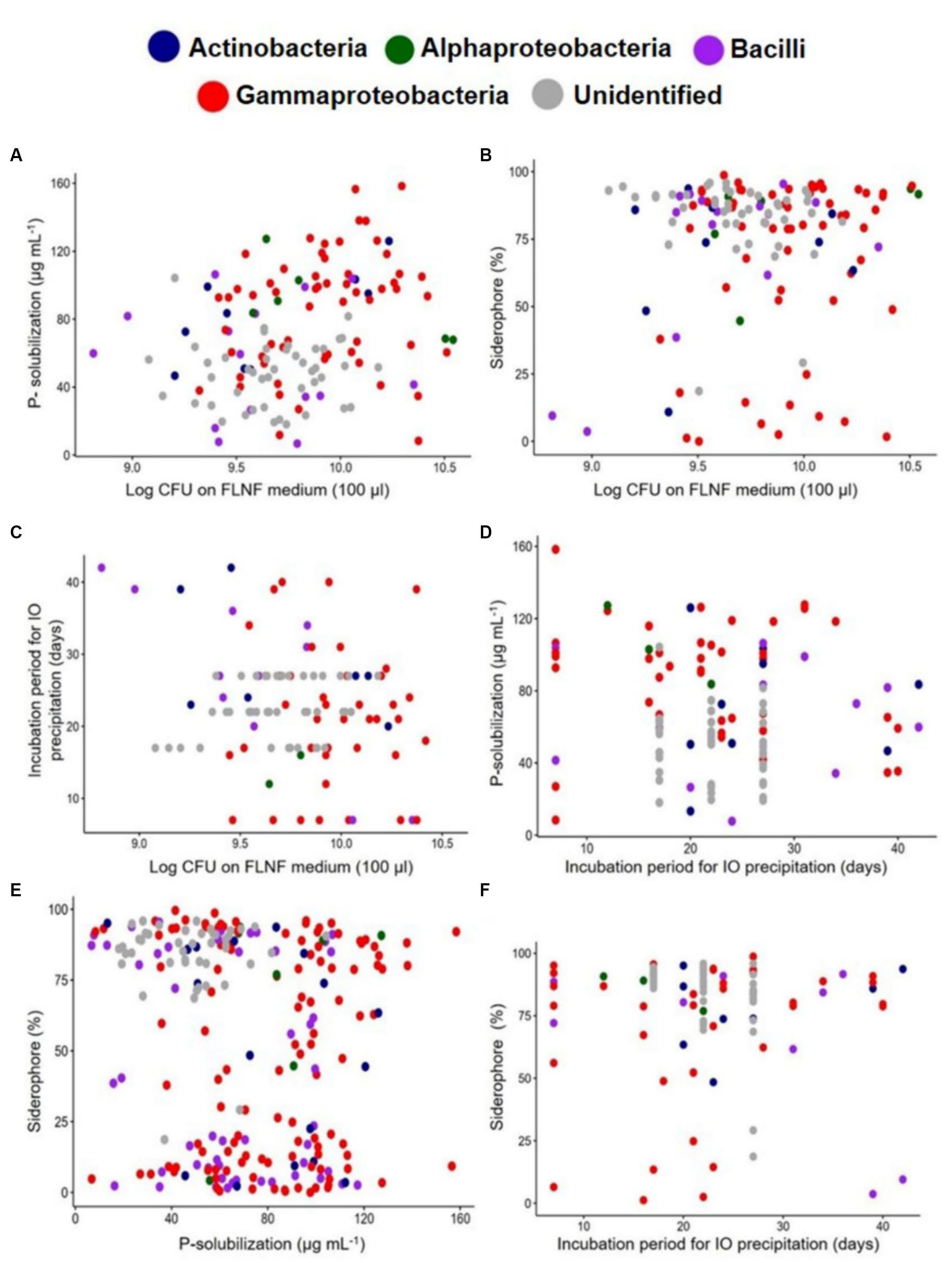
Scatter plot of individual bacterial isolates designated class-wise with different colored dots depicting BNF-related multi-functions. **(A)** Log_10_CFU on FLNF medium and the P-solubilization, *n* = 135. **(B)** Log_10_CFU on FLNF medium and Siderophore%, *n* = 135. **(C)** Log_10_CFU on FLNF medium and Incubation period (days) for IO precipitation, *n* = 109. **(D)** The incubation period (days) for IO precipitation and P-solubilization, *n* = 110. **(E)** P-solubilization and Siderophore%, *n* = 235. **(F)** The incubation period for IO precipitation and Siderophore%, *n* = 110. Each dot represents an individual isolate.

Scatter plots were constructed of pairs of BNF-facilitating functions (Figure 6) for different smaller subsets of total isolates (*n* = 135, 109, 110, and 235) of the FLNFM- and IOM-selected isolates (Figures 6A–F). Most of the FLNFM- or IOM-selected Gammaproteobacteria exhibited high values for all four functions under laboratory conditions (Figures 6A–F). This visualization might appear biased because, out of the 190 accessioned isolates in the analysis, 116 (~61%) were Gammaproteobacteria (compared to 50 were Bacilli, ~26.3%; 17 were Actinobacteria, ~9%; and 7 were Alphaproteobacteria, ~3.7%).

**Figure 7:**
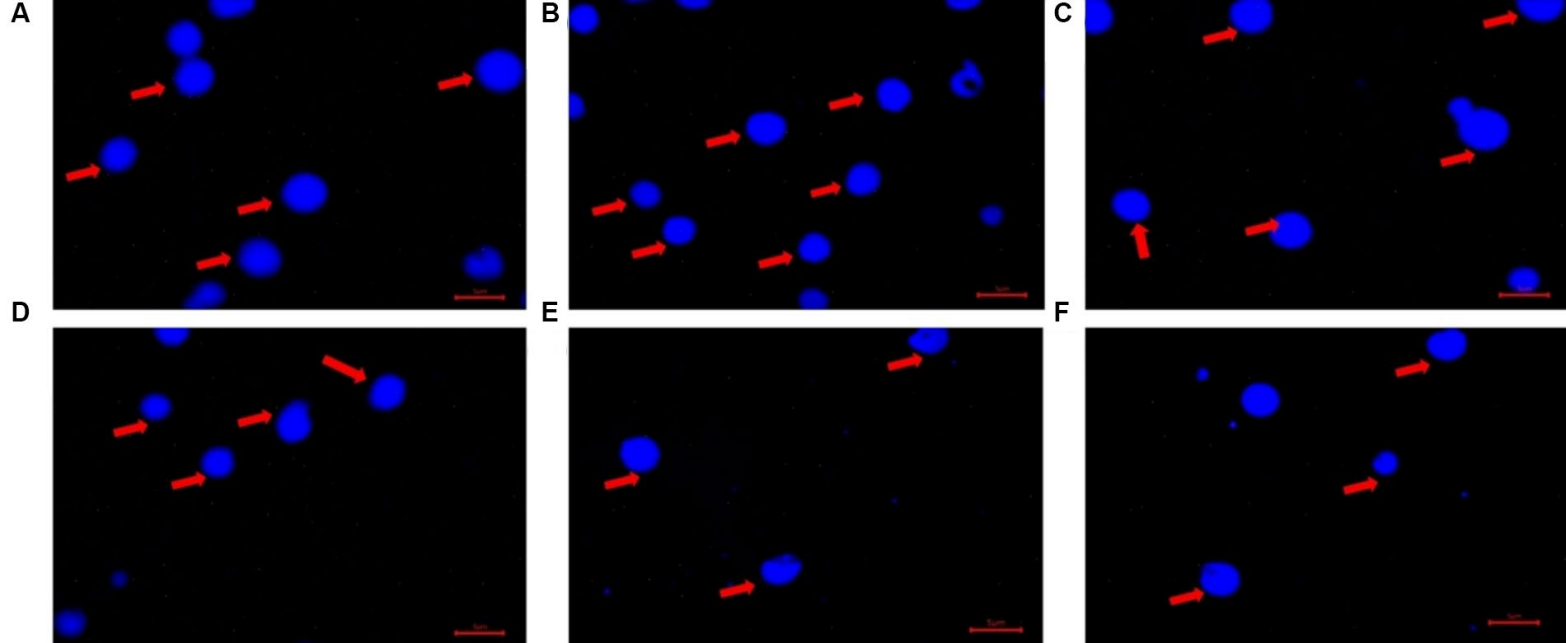
The DAPI stained images of extracted Poly-P granules under a fluorescence microscope, excitation at 370 nm, emission at 526 nm. **(A)** Stock Poly-P granules (2 μg μl^−1^) sodium phosphate glass 45 (Sigma Aldrich). **(B)** Extracted Poly-P (0.0410 μg μl^−1^) from *Acinetobacter johnsonii* strain SIO1 grown under anoxic condition. **(C)** Extracted Poly-P (0.0216 μg μl^−1^) from *Escherichia coli* strain K12 PR 1031 grown under anoxic condition (control). **(D)** Extracted Poly-P (0.0158 μg μl^−1^) from *Pseudomonas fluorescens* strain AFN1 grown under aerobic condition. **(E)** Extracted Poly-P (0.0154 μg μl^−1^) from *Staphylococcus epidermis* strain DAL6 grown under aerobic condition. **(F)** Extracted Poly-P (0.0137 μg μl^−1^) from *Serratia marcescens* strain RSO8 grown under anoxic condition. Blue spots are DAPI stained Poly-P granules in aggregated forms as observed both from stock Poly-P **(A)** and extracted Poly-P from bacterial isolates **(B–F)**, indicated with red arrows.

On the same scatter plots, 75% confidence ellipses were individually added for FLNFM- and IOM-selected isolates, with the outliers outside the ellipses (Supplementary Figures S3A–F). Each FLNFM-selected isolates’ ellipse was wider and largely overlapped the corresponding IOM-selected isolates’ ellipse (Supplementary Figures S3A–F). Regarding log_10_CFU on FLNFM vs. P solubilization, more FLNFM-selected isolates exhibited high P solubilization (Supplementary Figure S3A). Regarding log_10_CFU on FLNFM vs. siderophore production, more IOM-selected isolates exhibited high siderophore production but they had a wide range of log_10_CFU on FLNFM (Figure 7B). Regarding log_10_CFU on FLNFM vs. time to iron oxidation, more IOM-selected isolates exhibited moderate time to iron oxidation, while FLNFM-selected isolates exhibited wide variability (Supplementary Figure S3C). Regarding time to iron oxidation vs. P solubilization, most IOM-selected isolates exhibited moderate P solubilization and moderate time to iron oxidation, while most FLNFM-selected isolates exhibited high P solubilization and variable time to iron oxidation (Supplementary Figure S3D). Interestingly, regarding P solubilization vs. siderophore production, the IOM- and FLNFM-selected isolates formed two distinct clusters, with most of the former exhibiting low P solubilization and high siderophore production while the latter exhibited high P solubilization and low siderophore production (Supplementary Figure S3E). Regarding time to iron oxidation vs. siderophore production, again the FLNFM-selected isolates formed a wider ellipse, with wide ranges of siderophore production and time to iron oxidation. In contrast, the IOM-selected isolates’ ellipse was small and encompassed by the FLNFM-selected isolates’ ellipse, demonstrating higher siderophore production and moderate time to iron oxidation (Supplementary Figure S3F).

The same smaller subsets of total isolates (*n* = 135, 109, 110, and 235) of the FLNFM- and IOM-selected isolates were then subjected to Pearson’s correlation analysis in R (Supplementary Figure S4). There was a significant positive correlation (*R* = 0.28, *p* = 0.0012) between log_10_CFU on FLNFM and P solubilization (Supplementary Figure S4A), a non-significant and weak positive correlation (*R* = 0.091, *p* = 0.29) between log_10_CFU on FLNFM and siderophore production (Supplementary Figure S4B), and a significant negative correlation (*R* = −0.22, *p* = 0.019) between log_10_CFU on FLNFM and time to iron oxidation (Supplementary Figure S4C). There was a non-significant and weak negative correlation (*R* = −0.13, *p* = 0.18) between time to iron oxidation and P solubilization (Supplementary Figure S4D), a significant negative correlation (*R* = −0.16, *p* = 0.016) between P solubilization and siderophore production (Supplementary Figure S4E), and a non-significant and very weak negative correlation (*R* = −0.054, *p* = 0.58) between time to iron oxidation and siderophore production (Supplementary Figure S4F).

Based on these in-depth comparisons of the four BNF-facilitating functions between IOM- and FLNFM-selected isolates, the following clear trends are apparent: (a) FLNF with moderate-to-high log_10_CFU on FLNFM were efficient P-solubilizers, regardless of selection on FLNFM or IOM, (b) moderate-to-high log_10_CFU on FLNFM was associated with high siderophore production, again regardless of selection on FLNFM or IOM, (c) IOM selection yielded moderate-to-high log_10_CFU on FLNFM, moderate P-solubilizers, and moderate time to iron oxidation (<25 days), and (d) IOM selection was closely associated with high siderophore production (Supplementary Figures S3A–F, S4A–F). All these trends regarding co-occurrences of the four BNF-facilitating functions possessed by many of the 235 diazotrophic isolates, selected on stringent IOM and FLNFM under laboratory conditions, validated our hypothesis that heterotrophic FLNF with multi-metabolic BNF-facilitating functions were selected in hostile estuarine mangrove niches.

### 3.5 Comprehensive analyses of 24 FLNFM- and IOM-selected isolates from mangrove niches

Further analyses of multiple diverse metabolic functions (including the four major BNF-facilitating functions) were carried out under laboratory conditions for a subset of 24 heterotrophic, diazotrophic, accessioned bacterial isolates (11 Gammaproteobacteria, 7 Bacilli, 4 Actinobacteria, and 2 Alphaproteobacteria, belonging to 18 genera) that were isolated from the five estuarine mangrove niches considered in this study (CR_R_, M_R_, M_NR_, HNG_R_, and MRP_E_) (Supplementary Data). The isolates were *Brachybacterium aquaticum* CD6, *Curtobacterium* sp. PS25, *Glutamicibacter arilaitensis* CD22, *Micrococcus yunnanensis* APS6, *Aneurinibacillus* sp. MSO2, *Bacillus altitudinis* XYL1, *Bacillus licheniformis* DNB1, *Bacillus oceanisediminis* BCY3, *Bacillus* sp. SAN1, *Bacillus thuringiensis* CRL3, *Staphylococcus epidermis* DAL6, *Rhizobium* sp. FNF10, *Rhizobium* sp. FNF3, *Acinetobacter johnsonii* SIO1, *Acinetobacter* sp. SN3, *Aeromonas allosaccharophila* DAL2, *Citrobacter freundii* ERS1, *Enterobacter roggenkampii* RIO6, *Pantoea agglomerans* RPS2, *Pseudocitrobacter faecalis* HRR5, *Pseudomonas fluorescens* AFN1, *Serratia marcescens* RSO8, *Shewanella seohaensis* SSO7, and *Vibrio parahaemolyticus* ANI4.

All 24 of the isolates were positive for both P solubilization and the acetylene reduction assay for the functional nitrogenase (Supplementary Figures S5A,B). Including these 24 isolates, a total of 84 accessioned FLNFs selected on FLNFM repeatedly, were tested for ARA (Supplementary Data) that demonstrated production of nmol of ethylene in the range of 0–19.216 ± 0.23 per 24 h (observed RT range of ethylene was 3.945–4.0717 min). Some of the Gammaproteobacteria isolates exhibited very high P solubilization and acetylene reduction abilities (Supplementary Figure S5). The two Alphaproteobacteria (*Rhizobium*) isolates exhibited very high log_10_CFU on FLNFM, while the Gammaproteobacteria, Bacilli, and Actinobacteria exhibited wide ranges (Supplementary Figure S6A), displaying high log_10_CFU on FLNFM was not always overlapping with high values from ARA assays. Most of the 24 isolates were high siderophore producers (>75%) (Supplementary Figure S6B). There were 6 non-IOB, with no visible iron oxide precipitate forming within the maximum 42 days of observation (Supplementary Data). The remaining 18 isolates formed visible iron oxide precipitate under aerobic (DO of 6.85 mg L^−1^) and/or anoxic (DO of 0.05 mg L^−1^) conditions (Supplementary Figure S7) and under both aerobic and anoxic conditions after 100 mg L^−1^ NO_3_-N was added to the stringent minimal IOM at pH 6.0 to cause nitrate-dependent oxidation of ferrous iron under both aerobic and anaerobic conditions, which has been reported for DB (Straub et al., 2001).

The iron oxide precipitate formation was found to be coupled to denitrification, with all 24 isolates being good denitrifiers (Supplementary Figure S8B). The 24 isolates were evaluated for soluble P uptake and denitrification (Supplementary Figures S8A,B). Soluble P uptake was generally lower (below ~20%) while denitrification was mostly high (~70–99% over 24 h) under both aerobic (DO of 6.78 mg L^−1^) and anoxic (DO of 0.07 mg L^−1^) conditions. Most of the 24 isolates (except 1–2 isolates) had similar soluble P uptake and denitrification under aerobic vs. anoxic conditions (Supplementary Data; Supplementary Figures S8A,B). Two control laboratory strains (*E. coli* K12 ER2925 and *E. coli* K12 PR1031) were utilized for the P uptake and denitrification experiments, as these are known denitrifiers with very low P uptake ability (Mukherjee et al., 2019).

After 72 h of P uptake, accumulated poly-P was extracted from the isolates and quantified. The two Actinobacteria isolates (*Brachybacterium aquaticum* CD6 and *Curtobacterium* sp. PS25) were good poly-P accumulators (~0.4–0.5 mg g^−1^ of dry weight of cells), while the remainder of the isolates exhibited comparatively lower poly-P accumulation (<0.2 mg g^−1^ of dry weight of cells) (Supplementary Figure S8C). Most isolates (except 1–2) showed a negligible difference in poly-P accumulation under aerobic vs. anoxic conditions (Supplementary Figure S8C). Under both aerobic and anoxic conditions, most isolates (except for the two Actinobacteria isolates) did not exhibit concurrence between efficient P uptake and greater poly-P accumulation (Supplementary Figures S9A,B). Extracted poly-P granules from the 24 isolates were stained with DAPI and visualized under a fluorescent microscope after 72 h of incubation (Figure 7). To visually confirm the appearance of the accumulated poly-P granules, they were compared to the appearance of a standard poly-P compound (sodium phosphate glass 45; Sigma Aldrich), which was used to prepare a standard curve.

We hypothesized that this metabolic versatility of diazotrophic denitrifiers is equivalent to “cryptic BNF.” The metabolic versatility allows the nitrogenase to function as a hydrogenase to yield H_2_, which is used to reduce NO_3_^−^ to N_2_; this N_2_ can be fixed by the nitrogenase, making the whole internal BNF process undetectable (cryptic BNF). Thus, these diazotrophs reduce the high NO_3_^−^ concentration dissolved in O_2_-minimum zones (OMZs) of oceans and use nitrogenase as a free hydrogen producer, while cryptic BNF and denitrification both can concur (Reeder, 2021). The intriguing set of manifestations (such as high diversity of diazotrophs with low BNF in OMZs of oceans, positive correlations of NO_3_^−^ and NH_4_^+^ with diazotrophic operational taxonomic units in OMZs, and assimilation of NO_3_^−^ being energetically unfavorable at low O_2_) points to at least a spatial coupling between denitrification and diazotrophs’ habitats in OMZs, if not a coupling of denitrification and BNF in OMZs (Reeder et al., 2022).

Poly-P granules (volutin granules) are straight-chain polymers of tens to hundreds of phosphate residues linked with energy-intensive phosphoanhydride bonds. Microorganisms store excess cellular soluble P in this insoluble form, which allows them to use poly-P as a nutrient and an energy supply under P-deficient conditions. Polyphosphate kinases and exopolyphosphatases are key enzymes, located in the same operon, that are responsible for poly-P synthesis/storage and energy release via poly-P degradation, respectively (Achbergerová and Nahálka, 2011). The biological component in P cycling is based on C/N/P ratio of cells, where this C/N/P ratio is influenced by Poly-P storage and degradation (Akbari et al., 2021). Poly-P storage in diazotroph bacterial cells as observed (Figure 7), could be an important strategy in stringent mangrove niches like Sundarbans for their survival, an alternative source of energy to perform metabolic functions under P-limitation. Similar observations by other researchers also established an abundance of P-accumulating bacteria in adverse mangrove niches. When 134 sludge samples from the mangrove wetlands in Dongzhaigang, Hainan Island in China were screened for polyphosphate accumulation, 185 isolates of polyphosphate accumulating organisms (PAOs) were obtained with 42 PAOs exhibiting 20–80% P-removal, which signified phosphorus removal as a natural mechanism preventing eutrophication in water bodies (Wu et al., 2016). *Spartina alterniflora* invasion was found to be associated with the simultaneous significant abundance of sulfate reduction genes, sulfur oxidation genes, and polyphosphate kinase (*ppk*) genes, at 25–50 cm depth of mangrove sediment, establishing a strong positive correlation between P and S co-bioconversion, as a unique adaptive trait of avoiding excessive P deposition in marshes causing eutrophication via sulfur metabolism-linked biological P removal (Mo et al., 2023). An obvious metabolic coupling between BNF and P-accumulation/solubilization was observed in *S. alterniflora-*invaded subtropical mangrove niches in the Beibu Gulf of China (Zhang et al., 2022). One hundred and eight reconstructed genomes associated with N_2_ fixation and P-accumulation/solubilization, assigned majorly Gammaproteobacteria and Deltaproteobacteria for this N-P coordinated metabolism. In addition, N_2_ fixation, sulfate reduction, iron reduction to a form unable to bind P, and P-solubilization, thus increasing available P in sediment, all attributes displayed by the same bacterial genome, corroborated coupling between the C, N, P, S, and iron cycles mediated by these organisms in mangrove sediments.

The presence of poly-P in diazotrophs coupled with denitrification might be considered an additional adaptive strategy for heterotrophic diazotrophs to conduct BNF in P-deficient mangrove niches. It has long been known that there are many aerobic bacterial denitrifiers (such as *Acinetobacter* spp., *Pseudomonas* spp., *Agrobacterium* spp., *Pasteurella* spp., *Sphingomonas* spp., *Hydrogenophaga* spp., *Citrobacter* spp., and *Xanthomonas* spp.), many of which are diazotrophs. Aerobic bacterial denitrifiers can accumulate large amounts of poly-P (~0.3–27 mg g^−1^ of dry weight), confirming that poly-P accumulation and denitrification can be carried out by the same bacteria (Jørgensen and Pauli, 1995). In addition, *Rhizobium leguminosarum*, an important symbiotic diazotroph, was found to be a good poly-P accumulator, and poly-P storage and degradation efficiently contributed to symbiotic BNF (Akbari et al., 2021). *Pseudomonas stutzeri* ADP-19, a diazotroph was characterized by the simultaneous removal of nitrogen and phosphorus by a novel aerobic denitrifying phosphorus-accumulating mechanism leading to excessive poly-P accumulation (Li et al., 2021). Poly-P granules were also visible in all growth phases of *Rhizobium fredii* KR23, another symbiotic diazotroph, when labeled with anti-lipopolysaccharide-gold complex (Yang and Lin, 1998). The diazotrophic aerobic denitrifiers *Pseudomonas stutzeri* YG-24 and *Agrobacterium* sp. LAD9 exhibited simultaneous aerobic denitrification and P uptake, to provide energy for heterotrophic BNF (Gaimster et al., 2018). Furthermore, 25 aerobic denitrifier P-accumulator bacterial strains that use NO_3_^-^-N as an electron acceptor exhibited denitrification with simultaneous uptake of excessive P under aerobic conditions (Li et al., 2019). Many of these strains belonged to five genera (*Aeromonas*, *Citrobacter*, *Pseudomonas*, *Acinetobacter*, and *Delftia*), many of which are known diazotrophs (Li et al., 2019). They had a P uptake rate of up to 82.32% and a simultaneous denitrification rate of up to 73.73% (Li et al., 2019).

The association of BNF with other metabolic pathways was evident from many other earlier researches. In the mangrove sediment microbiome, the high frequency of Chloroflexi and Nitrospirae was justified as an adaptive characteristic of sulfide-rich mangrove habitat, demonstrating the coupling of carbon, nitrogen, and sulfur cycles (Lin et al., 2019). While the N-cycle operates via ammonification and DNRA to NH_4_^+^-N, to conserve N, it is linked to dissimilatory sulfate reduction and polysulfide formation, and 36 bacterial species were identified, as “potential biogeochemical linkers,” 40 and 15% belonging to Deltaproteobacteria and Gammaproteobacteria, respectively (Lin et al., 2019). The coupling of soil N and P was demonstrated by N addition in Songnen Meadow Steppe in northeast China, increasing available N, and the N:P ratio, however decreasing considerably total P and available P (Zhang et al., 2013). Apart from sulfur-reducing bacteria (SRB), sulfur-oxidizing bacteria (SOB) were described as integral components of mangrove sediment microbiome, and SOB like *Thiobacillus denitrificans* can utilize nitrate as an oxidant in deeper sediments where oxygen was limited (Kashif et al., 2023). The abundance of two well-known diazotroph genera *Pseudomonas* and *Acinetobacter* spp. was reported in rhizosphere soil with high P accumulation and a strong correlation with P-accumulating trait with high P-solubilization and siderophore production (Srivastava et al., 2022). Four PGPR bacterial strains such as *Providencia rettgeri* P2, *Advenella incenata* P4, *Acinetobacter calcoaceticus* P19, and *Serratia plymuthica* P35 were reported as good P-solubilizers with simultaneous BNF potential (Li et al., 2020). Evaluation of three PGP strains (*Pantoa* HRP2, *Enterobacter* SSP2, *Pseudomonas* JRP22) of P-solubilizers promoting the growth of Chinese fir seedlings, displayed simultaneous nitrogenase activity and high siderophore production ability (Chen et al., 2021).

Despite this culture-based approach established 235 isolates as heterotrophic FLNF out of the 299 isolates, most of these FNF species identified in our NGS analyses, demonstrated a very low relative abundance for them in mangrove-related rhizospheres (Supplementary Data). One possible explanation might be the unfavorable resource-limited stringent niches and competitive multi-species/multi-trophic interactions prevailing in these estuarine niches challenging the survivability and growth of the resident FNF community (Fierer and Lennon, 2011; Faust and Raes, 2012; Weiss et al., 2016). In laboratory-based cultures under optimum conditions and adequate resources, in pure form, in the absence of any competition, these isolates exhibited the highest fitness to demonstrate growth on FLNFM and subsequently yielded positive results in ARA confirmatory test (Acetylene Reduction Assay).

Our comprehensive analysis of the subset of 24 heterotrophic diazotrophs from hostile high-salinity estuarine mangrove niches in the Indian Sundarbans confirmed that in addition to demonstrating the four BNF-facilitating functions, namely, log_10_CFU on FLNFM (along with acetylene reduction assay for the function of nitrogenase), P solubilization, siderophore production, and iron oxidation (visualized by iron oxide formation), these isolates are capable of denitrification coupled to BNF under both anoxic and aerobic conditions, soluble P uptake, and efficient poly-P accumulation. Co-occurrence of all these diverse metabolic traits by the same heterotrophic diazotrophs indicates the facilitation of functional BNF in hostile estuarine mangrove environments.

## 4 Conclusion

A new ecological concept posits that all microbes evolve in a particular niche that shapes their microbiome, with microbes adopting either multidimensional specialization or multidimensional generalization (Hernandez et al., 2023). From the present lab-based study, it can be concluded that the stringent and narrow parameters of various intertidal estuarine mangrove niches drove the multidimensional specialization of the heterotrophic diazotroph communities. Multidimensional specialists face more environmental constraints during their evolution and are less resilient and less common compared to their generalist counterparts (Hernandez et al., 2023). The extreme nature of the estuarine mangrove-related rhizospheric sediment parameters (related to pH, salinity, temperature, soil texture, dynamic relative abundances of bacterial taxa, deficiencies in N, P, and Fe, presence of sulfides, and anoxicity) structured the heterotrophic diazotroph communities, which exhibited diverse metabolic functions that aid BNF. The culturable heterotrophic diazotrophs specialized in diazotrophy concurrent with P solubilization, iron oxidation, siderophore production, denitrification, P uptake, and poly-P accumulation, gradually developing a multidimensional specialization. The results established that the selection of the heterotrophic diazotrophs on stringent IOM was similarly pertinent (in terms of selecting isolates with many efficient metabolic functions) to the selection on FLNFM. These selected culturable diazotrophs may act as potential biogeochemical linkers, mediating coupled C, N, P, S, and Fe cycling as an adaptive strategy, which was also validated by earlier researchers from large-scale structured genomic/metagenomic data analyses exhibiting co-occurrence of respective functional genes in resident microbes/mangrove sediments. The abovementioned metabolic functions that aid BNF in hostile mangrove niches may promote mangrove growth if degraded nutrient-deficient mangrove rhizospheres were enriched with these diazotrophic multidimensional specialists as part of mangrove restoration initiatives. These diazotrophs could also be evaluated as prospective biofertilizers for agriculture in near-mangrove cultivated lands.

## Data availability statement

The datasets presented in this study can be found in online repositories. The names of the repository/repositories and accession number(s) can be found in the article/Supplementary material.

## Author contributions

SM: Data curation, Formal analysis, Investigation, Methodology, Visualization, Writing – review & editing. BB: Data curation, Formal analysis, Investigation, Methodology, Visualization, Writing – review & editing. RC: Data curation, Formal analysis, Investigation, Visualization, Writing – review & editing. RS: Data curation, Formal analysis, Investigation, Methodology, Software, Validation, Visualization, Writing – original draft, Writing – review & editing. AM: Investigation, Methodology, Software, Validation, Writing – original draft, Writing – review & editing. HK: Investigation, Methodology, Software, Validation, Writing – original draft, Writing – review & editing. CG: Investigation, Methodology, Validation, Writing – review & editing. AG: Data curation, Methodology, Formal analysis, Writing – review & editing. SS: Investigation, Methodology, Validation, Writing – original draft, Writing – review & editing. MB: Investigation, Methodology, Writing – review & editing. CM: Conceptualization, Investigation, Methodology, Validation, Writing – review & editing. ID: Investigation, Methodology, Validation, Writing – review & editing. SB: Funding acquisition, Resources, Supervision, Visualization, Writing – review & editing. MM: Methodology, Supervision, Visualization, Writing – review & editing. KR: Conceptualization, Formal analysis, Funding acquisition, Investigation, Project administration, Resources, Supervision, Validation, Visualization, Writing – original draft, Writing – review & editing.

## Footnotes

^1^ https://rsis.ramsar.org/ris/2370?language=en

^2^ https://www.unesco.org/en/mab/sunderban

^3^ https://sundarbantigerreserve.org/
