# Supplementary material for: Estuarine mangrove niches select cultivable heterotrophic diazotrophs with diverse metabolic potentials—a prospective cross-dialog for functional diazotrophy
Source: Front Microbiol. 2024 May 24;15:1324188. doi: 10.3389/fmicb.2024.1324188 (PMC11174608; doi:10.3389/fmicb.2024.1324188)
Supplement: Supplementary file 2 [file Data_Sheet_1.PDF]

# Supplementary Information

# Estuarine mangrove niches select cultivable heterotrophic diazotrophs with diverse metabolic potentials - A prospective cross-dialogue for functional diazotrophy

**Sumana Mondal[1ǂ], Biswajit Biswas[1,3ǂ], Rajojit Chowdhury[1,4ǂ], Rudranil Sengupta[1ǂ], Anup Mandal[1], Hemendra Nath Kotal[1], Chayan Kumar Giri[1], Anjali Ghosh[1], Subhajit Saha[1], Mst. Momtaj Begam[1,5], Chandan Mukherjee[1,6], Ipsita Das[1], Sandip Kumar Basak[2], Mahashweta Mitra Ghosh[3], Krishna Ray[1]***

[1]Environmental Biotechnology Group, Department of Botany, West Bengal State University, Berunanpukuria, Malikapur, Barasat, Kolkata 700126, India

[2]Department of Botany, Sarat Centenary College, Dhaniakhali, Hooghly 712302, West Bengal, India

[3]Department of Microbiology, St. Xavier's College (Autonomous), 30, Mother Teresa Sarani Kolkata 700016, West Bengal, India

[4]Department of Botany, Sree Chaitanya College, Habra, Prafullanagar, 24 Pgs(N) 743268, West Bengal, India

[5]Department of Botany, Kalimpong College, Rinkinpong Rd, Kalimpong Khasmahal, Kalimpong, West Bengal 734301, India

[6]School of Biological and Life Sciences, Galgotias University, Greater Noida, Gautam Buddh Nagar, Uttar Pradesh 203201, India

ǂ Authors contributed equally as joint first authors

*** Correspondence:**

Dr. Krishna Ray (She/Her)
kray91@gmail.com

ORCID ID: KR, 0000-0002-1893-5993

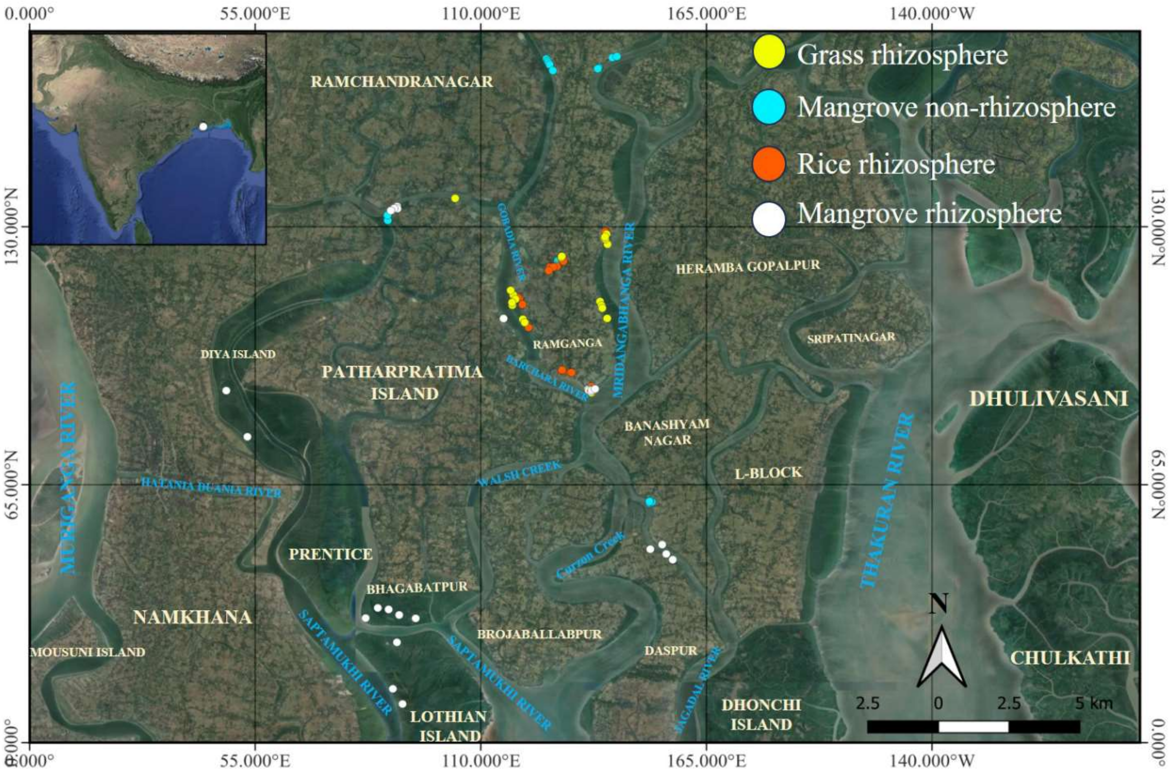


Figure S1. The map displays the locations of study sites as well as the collection points of soil sediment cores, onsite hydrology assessment, and collection of root/pneumatophore samples across the western part of the Indian Sundarbans. The different rhizospheric/non-rhizospheric sites are color-coded for easy identification: yellow represents the halophytic native grass rhizospheres ($HNG_R$), aqua represents the mangrove non-rhizospheres ($M_{NR}$), orange represents the cultivated rice rhizospheres ($CR_R$), and white represents the mangrove rhizospheres ($M_R$) as referred in the main manuscript. The sampling sites for root/pneumatophores overlap majorly with $HNG_R$ and $M_R$ sites. All the study sites are located across intertidal shorelines of the study regions and cultivated rice fields are located just juxtaposed to shoreline mangrove fringes.

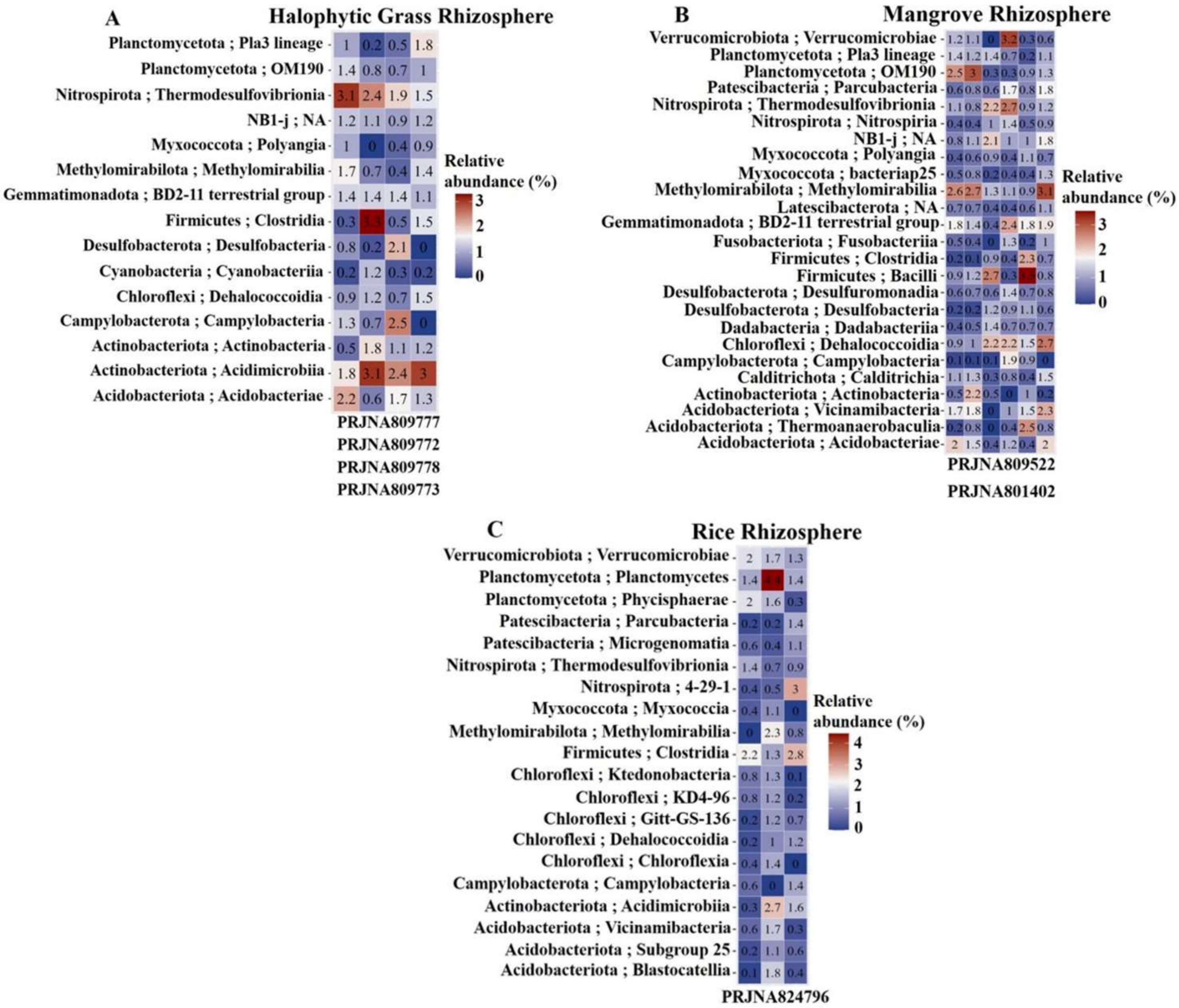


Figure S2. Three heatmaps displaying different bacterial classes (Y-axis) from 3 different mangrove-related rhizospheres from Indian Sundarbans (labeled in columns). The bacterial classes within 5% to 1% relative abundance level across all Biosamples have been demonstrated. (A) Halophytic Grass Rhizosphere. (B) Mangrove Rhizosphere. (C) Cultivated Rice Rhizosphere. The heatmaps were built on different Biosample reads under different NCBI Bioprojects involving mangrove-related rhizosphere types (X-axis).

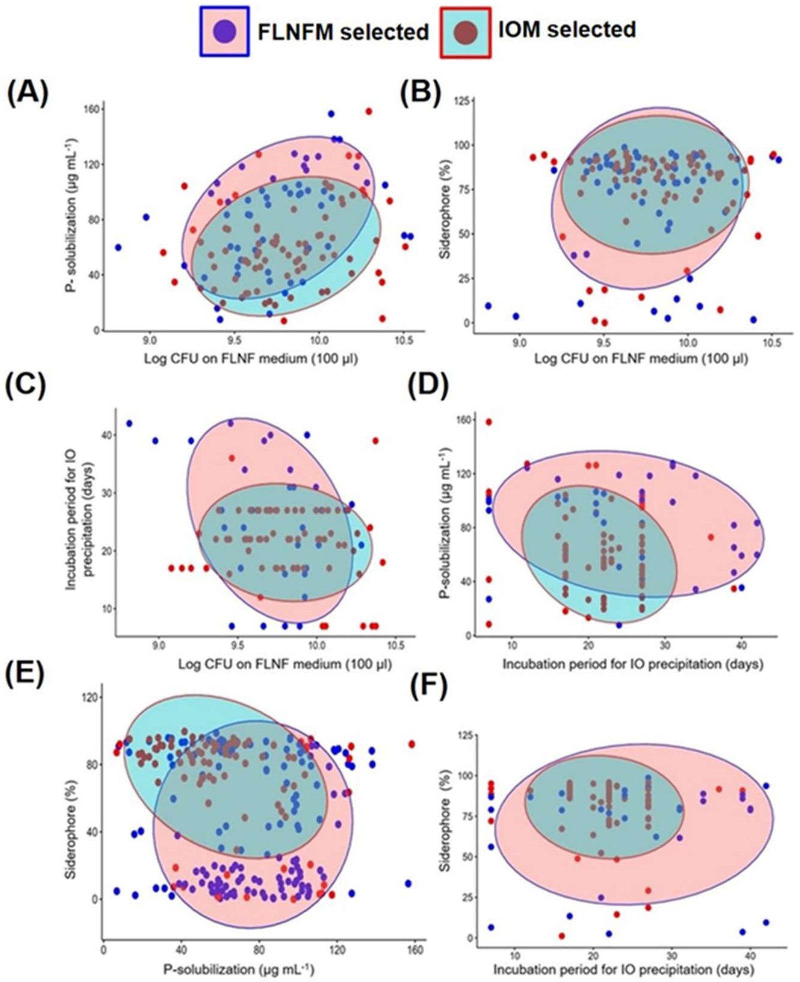


Figure S3. Scatter plot with confidence ellipse between BNF-related multi-functions of bacterial isolates selected from two different selection medium i.e. FLNFM and IOM. (A) $Log_{10}$CFU on FLNF medium and the P-solubilization, n=135. (B) $Log_{10}$CFU on FLNF medium and Siderophore%, n=135. (C) $Log_{10}$CFU on FLNF medium and Incubation period (days) for IO precipitation, n=109. (D) Incubation period (days) for IO precipitation and P-solubilization, n=110. (E) P-solubilization and Siderophore%, n=235. (F) Incubation period for IO precipitation and Siderophore%, n=110. Each dot represents an individual isolate. The individual ellipse shows the cluster of isolates satisfying 75% confidence score of the relations.

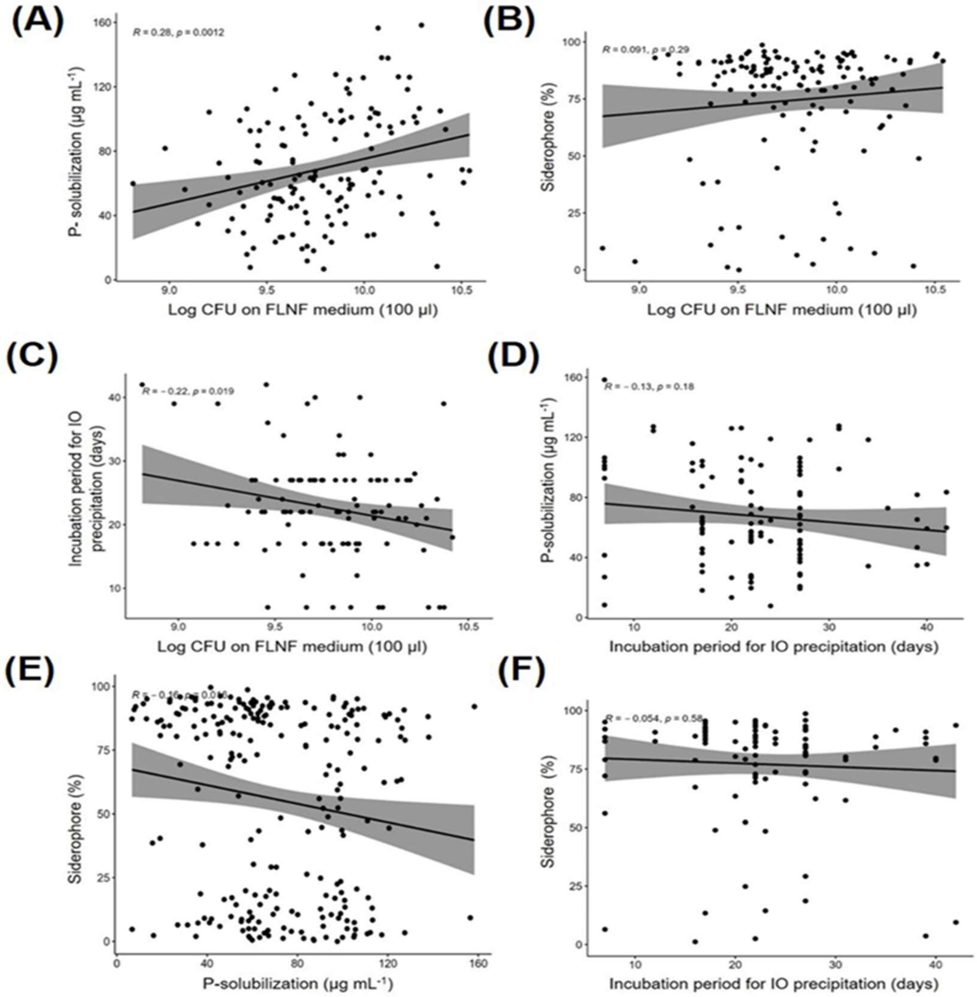


Figure S4. Pearson's Correlations depicted between BNF related multi-functions. (A) $Log_{10}$CFU on FLNF medium and the P-solubilization, n=135. (B) $Log_{10}$CFU on FLNF medium and Siderophore%, n=135. (C) $Log_{10}$CFU on FLNF medium and Incubation period (days) for IO precipitation, n=109. (D) Incubation period (days) for IO precipitation and P-solubilization, n=110. (E) P-solubilization and Siderophore%, n=235. (F) Incubation period for IO precipitation and Siderophore%, n=110. Each dot represents an individual isolate. The solid line indicates the linear regression of the variables. The gray areas indicate the 95% confidence intervals. Pearson correlation (R) and the statistical significance (*p*) are also shown.

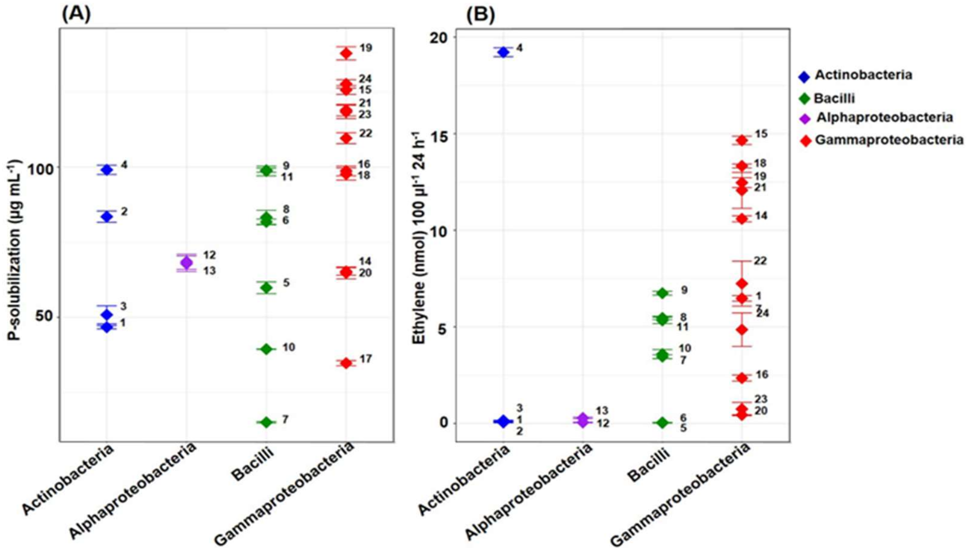


Figure S5. Dot Plot representing assay of functions facilitating BNF from 24 bacterial strains represented class-wise as observed under laboratory conditions. (A) P-solubilization (µg $mL^{-1}$). (B) Ethylene production in (nmol) 100 $\mu L^{-1}$ 24 $h^{-1}$ in ARA. Standard error bars given for 3 biological replicate experiments. 1. *Brachybacterium aquaticum* strain CD6, 2. *Curtobacterium* sp. strain PS25, 3. *Glutamicibacter arilaitensis* strain CD22, 4. *Micrococcus yunnanensis* strain APS6, 5. *Aneurinibacillus* sp.strain MSO2, 6. *Bacillus altitudinis* strain XYL1, 7. *Bacillus licheniformis* strain DNB1, 8. *Bacillus oceanisediminis* strain BCY3, 9. *Bacillus* sp. strain SAN1, 10. *Bacillus thuringiensis* strain CRL3, 11. *Staphylococcus epidermis* strain DAL6, 12. *Rhizobium* sp. strain FNF10, 13. *Rhizobium* sp. strain FNF3, 14. *Acinetobacter johnsonii* strain SIO1, 15. *Acinetobacter* sp. strain SN3, 16. *Aeromonas allosaccharophila* strain DAL2, 17. *Citrobacter freundii* strain ERS1, 18. *Enterobacter roggenkampii* strain RIO6, 19. *Pantoea agglomerans* strain RPS2, 20. *Pseudocitrobacter faecalis* strain HRR5, 21. *Pseudomonas fluorescens* strain AFN1, 22. *Serratia marcescens* strain RSO8, 23. *Shewanella seohaensis* strain SSO7, 24. *Vibrio parahaemolyticus* strain ANI4.

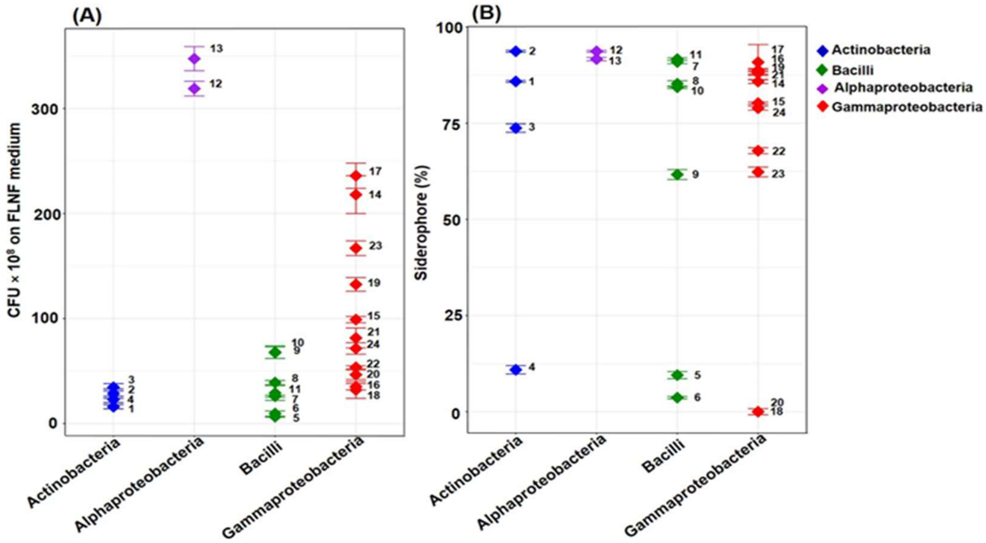


Figure S6. Dot Plot representing assay of functions facilitating BNF from 24 bacterial strains represented class-wise as observed under laboratory conditions. (A) CFU*$10^8$ 100 µ$L^{-1}$ on FLNF medium. (B) Siderophore production%. Standard error bars given for 3 biological replicate experiments. 1. *Brachybacterium aquaticum* strain CD6, 2. *Curtobacterium* sp. strain PS25, 3. *Glutamicibacter arilaitensis* strain CD22, 4. *Micrococcus yunnanensis* strain APS6, 5. *Aneurinibacillus* sp.strain MSO2, 6. *Bacillus altitudinis* strain XYL1, 7. *Bacillus licheniformis* strain DNB1, 8. *Bacillus oceanisediminis* strain BCY3, 9. *Bacillus* sp. strain SAN1, 10. *Bacillus thuringiensis* strain CRL3, 11. *Staphylococcus epidermis* strain DAL6, 12. *Rhizobium* sp. strain FNF10, 13. *Rhizobium* sp. strain FNF3, 14. *Acinetobacter johnsonii* strain SIO1, 15. *Acinetobacter* sp. strain SN3, 16. *Aeromonas allosaccharophila* strain DAL2, 17. *Citrobacter freundii* strain ERS1, 18. *Enterobacter roggenkampii* strain RIO6, 19. *Pantoea agglomerans* strain RPS2, 20. *Pseudocitrobacter faecalis* strain HRR5, 21. *Pseudomonas fluorescens* strain AFN1, 22. *Serratia marcescens* strain RSO8, 23. *Shewanella seohaensis* strain SSO7, 24. *Vibrio parahaemolyticus* strain ANI4

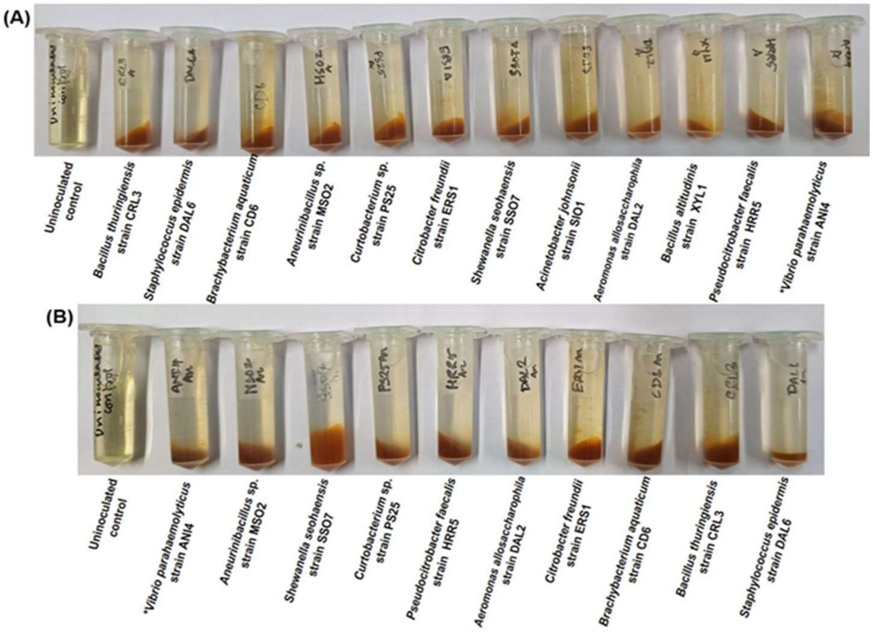


Figure S7. Images showing the precipitation of iron oxidation (brick red color) by different bacterial isolates with respect to uninoculated control. (A) Under aerobic condition with DO of 6.85 mg $L^{-1}$. (B) Under anoxic condition with DO of 0.05 mg $L^{-1}$.

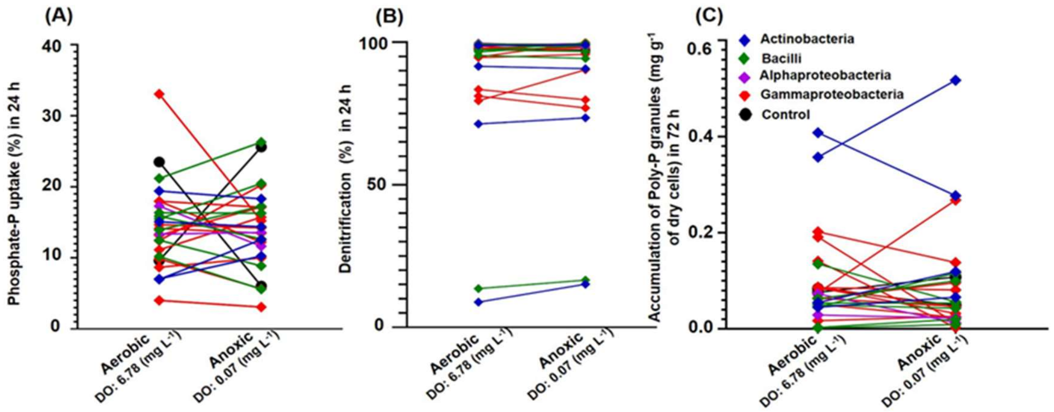


Figure S8. The paired dot diagram showing the comparisons of related functions facilitating BNF from 24 bacterial strains represented class-wise as observed under laboratory conditions. (A) Percentage of phosphate-P uptake potential in 24 h. (B) Percentage of denitrification potential in 24 h. (C) Accumulation of Poly-P granules in mg $g^{-1}$ of dry cells in 72 hours. Two controls (black dots) are for *E. coli* K12 ER2925 and *E. coli* K12 PR1031. The lines join the relative positions of the same bacterial isolates represented as class-wise colored dots in both aerobic and anoxic conditions from 3 biological replicate experiments.

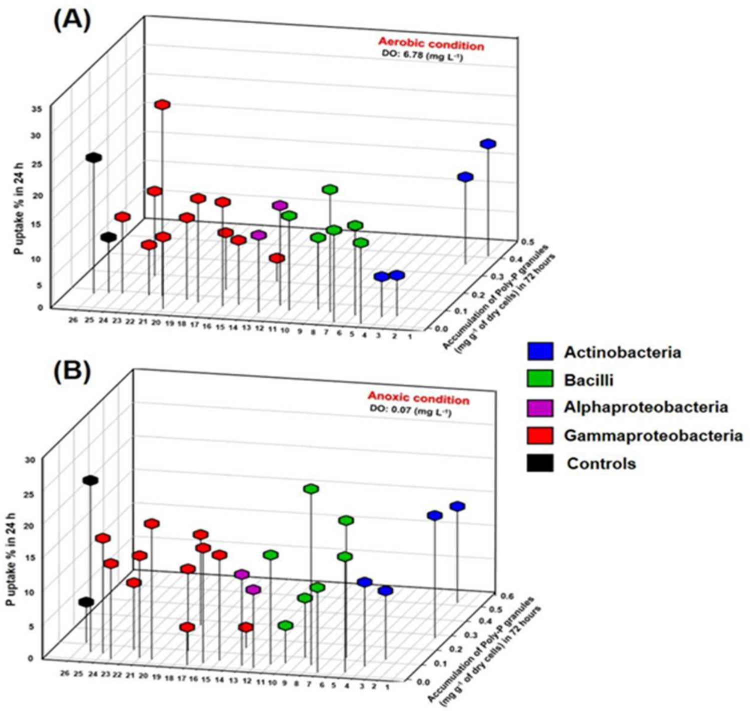


Figure S9. The 3D figure showing comparisons of percentage of P-uptake in 24 h (Y-axis) and the accumulation of poly-P granules in mg $g^{-1}$ of dry cells after 72 h (Z-axis) across 24 bacterial isolates belonging to 4 classes with two controls (black dots) (X-axis). (A) Aerobic condition with DO of 6.78 mg $L^{-1}$. (B) Anoxic condition with DO of 0.07 mg $L^{-1}$. Results of 3 biological replicate experiments are represented. Bacterial strains represented along X-axis are as follows:

1. *Brachybacterium aquaticum* strain CD6, 2. *Curtobacterium* sp. strain PS25, 3. *Glutamicibacter arilaitensis* strain CD22, 4. *Micrococcus yunnanensis* strain APS6, 5. *Aneurinibacillus* sp.strain MSO2, 6. *Bacillus altitudinis* strain XYL1, 7. *Bacillus licheniformis* strain DNB1, 8. *Bacillus oceanisediminis* strain BCY3, 9. *Bacillus* sp. strain SAN1, 10. *Bacillus thuringiensis* strain CRL3, 11. *Staphylococcus epidermis* strain DAL6, 12. *Rhizobium* sp. strain FNF10, 13. *Rhizobium* sp. strain FNF3, 14. *Acinetobacter johnsonii* strain SIO1, 15. *Acinetobacter* sp. strain SN3, 16. *Aeromonas allosaccharophila* strain DAL2, 17. *Citrobacter freundii* strain ERS1, 18. *Enterobacter roggenkampii* strain RIO6, 19. *Pantoea agglomerans* strain RPS2, 20. *Pseudocitrobacter faecalis* strain HRR5, 21. *Pseudomonas fluorescens* strain AFN1, 22. *Serratia marcescens* strain RSO8, 23. *Shewanella seohaensis* strain SSO7, 24. *Vibrio parahaemolyticus* strain ANI4, 25. *E. coli* K12 ER 2925, 26. *E. coli* K12 PR 1031.

**The calibration process of DO meter undertaken while conducting iron oxidation assays**

The estimation of dissolved oxygen (DO) of the YMI broth and bacterial cultures during the iron oxidation assay was conducted using the HANNA dissolved oxygen meter model HI9146-04. The DO probe used in the meter has a permeable polytetrafluoroethylene (PTFE) polymer membrane covering the polarographic sensor and a built-in thermistor for measuring temperature and its compensation. The PTFE membrane covering isolates the sensor from the sample solution but allows oxygen to pass through it. When a voltage is applied across the sensor, the passed oxygen through the membrane reacts with it and provides a reading for the measured temperature. The meter has a DO estimation range between 0.00 to 45.00 ppm, within 0.0 °C to 50 °C.

A two-step calibration process was conducted according to the manufacturer's instructions (https://img.izm.by/d/p70000132.manual.pdf) during the calibration of the DO meter. The instrument was initially calibrated in two points, a zero calibration (0.0%) and a slope calibration (100%). Before proceeding with the calibration the membrane cap of the probe was filled with HANNA HI7041S electrolyte solution, ensuring no air bubbles were present between the sensor and the membrane, and the probe was kept at rest for 15 minutes to ensure precise condition. Then the zero solution was prepared freshly by mixing HANNA HI7040-1 and HANNA HI7040-2 zero oxygen solution mixtures as per manufacturer's instruction. After that, the instrument was set for zero calibration, the DO probe was submerged into the zero oxygen solution and stirred gently for 2-3 minutes until the instrument showed 0.0% DO calibration on the screen. Once the instrument was set for zero calibration, the probe was removed from the zero solution, rinsed several times with distilled water to remove any remaining zero solution, and further set for slope calibration. The slope calibrations were carried out by holding the dry probe in the air for a few minutes until the instrument showed 100% DO calibration on the screen. During both calibrations, the readings were stable. Both calibrations were performed every week depending on the usage of the instrument and each time the electrolyte solution was replaced with a fresh one.

For the iron oxidation study, the initial DO levels of the uninoculated YMI broth with 100 mg $L^{-1}$ $NO_3^-$-N were measured by submerging the DO probe with the calibrated meter in the media and stirring gently until the DO readings in the instrument were stable. To create an anoxic environment, 1 cm thick liquid paraffin oil was poured after inoculation of the bacterial isolates on the top of each culture inside the tube. The reading of anoxic cultures was performed similarly by removing paraffin oil from the top before inserting the probe into the media. To check the $Fe^{2+}$ to $Fe^{3+}$ oxidation after different time intervals (days), 500 µL of broth was mixed with 1M ammonium thiocyanate solution in a 1:3 (V/V) ratio, if the brick red precipitate appeared within 15 minutes then the rest of the cultures were used to measure the DO level for both aerobic and anoxic conditions.

**Temperature ranges of sediment cores and range of hydrological criteria observed across the study sites**

The pre-monsoon (April-May) sediment temperatures varied across the study sites as 29.7 ± 0.35°C to 33.1 ± 0.09°C (at 15 cm depth) and 28 ± 0.17°C to 31.2 ± 0.74°C (at 30 cm depth); similarly, monsoon (August-September) sediment temperature profile ranged from 29.53 ± 0.07°C to 30.17 ± 0.12°C (at 15 cm depth) and 28.87 ± 0.09°C to 29.3 ± 0.17°C (at 30 cm depth), and post-monsoon (January-February) sediment cores exhibited the temperature variation from 20.37 ± 0.03°C to 24.23 ± 0.12°C (at 15 cm depth) and 19.15 ± 0.05°C to 23.1 ± 0.06°C (at 30 cm depth) (Supplementary Data).

The surface hydrological profile across sites (Supplementary Data) was observed as follows: at pre-monsoon (April-May), EC in between ~38.1-45.3 $dSm^{-1}$, pH ~7.3-8, DO ~6.8-7.15 ppm, temperature ~29.5-33.5°C, turbidity average ~44 NTU (range ~24-110 NTU), at monsoon (August-September), EC ~7.6-29.8 $dSm^{-1}$, pH ~7.4-8.2, DO ~6.2-7.1 ppm, temperature ~29.4-30.1°C, turbidity average ~78 NTU (range ~27.8-198.6 NTU) and at post-monsoon (January-February), EC ~25.7-40.8 $dSm^{-1}$, pH ~7.9-8.3, DO ~6.3-8.7 ppm, temperature ~20.4-24.3°C, turbidity average ~26 NTU (range ~12-55 NTU).

| **Name of the plant rhizospheres/study sites** | **Rhizosphere Types** | **BioProject ID** | **BioSample ID** | **SRA ID** | **Number of raw reads** | **Number of filtered reads** |
|---|---|---|---|---|---|---|
| *Myriostachya wightiana* | Halophytic Grass Rhizosphere | PRJNA809777 | SAMN26202177 | SRS12101062 | 162025 | 5676 |
| *Paspalum vaginatum* | Halophytic Grass Rhizosphere | PRJNA809772 | SAMN26202111 | SRS12114902 | 101512 | 3065 |
| *Porteresia coarctata* | Halophytic Grass Rhizosphere | PRJNA809778 | SAMN26202868 | SRS12101103 | 148892 | 5005 |
| *Sporobolus virginicus* | Halophytic Grass Rhizosphere | PRJNA809773 | SAMN26202168 | SRS12091782 | 143991 | 6846 |
| Ramganga (2022_1) | Mangrove Rhizosphere | PRJNA801402 | SAMN26255136 | SRS12140422 | 99523 | 10302 |
| Bhagbatpur | Mangrove Rhizosphere | PRJNA809522 | SAMN26183780 | SRS12086291 | 173655 | 29975 |
| Dashpur | Mangrove Rhizosphere | PRJNA809522 | SAMN26183779 | SRS12082328 | 176016 | 30359 |
| Ramganga (2022_2) | Mangrove Rhizosphere | PRJNA801402 | SAMN30504958 | SRS14826460 | 176180 | 36574 |
| Ramganga (2022_3) | Mangrove Rhizosphere | PRJNA801402 | SAMN30504960 | SRS14840947 | 170737 | 36263 |
| Ramganga 2021 | Mangrove Rhizosphere | PRJNA801402 | SAMN25342833 | SRS12077795 | 191293 | 4080 |

| | | | | | | |
|---|---|---|---|---|---|---|
| Rice field 1 | Cultivated Rice Rhizosphere | PRJNA824796 | SAMN27478637 | SRR18726894 | 259157 | 10726 |
| Rice field 2 | Cultivated Rice Rhizosphere | PRJNA824796 | SAMN30789297 | SRR21541564 | 178977 | 45119 |
| Rice field 3 | Cultivated Rice Rhizosphere | PRJNA824796 | SAMN30885898 | SRR21607174 | 185910 | 40741 |

Table S1. The Table represents the the type of rhizopsheres studied, NGS BioProject IDs, BioSample IDs and the SRA IDs as per the NCBI database along with respective raw reads and filtered reads generated per BioSample/SRA data.
